# Tetraphenylpyrimidine-Based AIEgens: Facile Preparation, Theoretical Investigation and Practical Application

**DOI:** 10.3390/molecules22101679

**Published:** 2017-10-10

**Authors:** Junkai Liu, Lingxiang Pan, Qian Peng, Anjun Qin

**Affiliations:** 1 State Key Laboratory of Luminescent Materials and Devices, South China University of Technology, Guangzhou 510640, China; ipowerjkl@outlook.com (J.L.); panlx2014@hotmail.com (L.P.); 2 Key Laboratory of Organic Solids, Beijing National Laboratory for Molecular Sciences, Institute of Chemistry, Chinese Academy of Sciences, Beijing 100190, China

**Keywords:** tetraphenylpyrimidine, aggregation-enhanced emission, restriction of intramolecular motion, structure-property relationship, explosive detection

## Abstract

Aggregation-induced emission (AIE) has become a hot research area and tremendous amounts of AIE-active luminogens (AIEgens) have been generated. To further promote the development of AIE, new AIEgens are highly desirable. Herein, new AIEgens based on tetraphenylpyrimidine (TPPM) are rationally designed according to the AIE mechanism of restriction of intramolecular motion, and facilely prepared under mild reaction conditions. The photophysical property of the generated TPPM, TPPM-4M and TPPM-4P are systematically investigated and the results show that they feature the aggregation-enhanced emission (AEE) characteristics. Theoretical study shows the high-frequency bending vibrations in the central pyrimidine ring of TPPM derivatives dominate the nonradiative decay channels. Thanks to the AEE feature, their aggregates can be used to detect explosives with super-amplification quenching effects, and the sensing ability is higher than typical AIE-active tetraphenylethene. It is anticipated that TPPM derivatives could serve as a new type of widely used AIEgen based on their facile preparation and good thermo-, photo- and chemostabilities.

## 1. Introduction

The research on aggregation-induced emission (AIE) is drawing increasing interest since it can solve the aggregation-caused quenching (ACQ) problem encountered by traditional organic luminophores. AIE-active luminogens (AIEgens) possess relatively high emission efficiency in the aggregate or solid states, which meets the requirements for practical applications in organic light-emitting diodes, optical waveguides, biosensors and bioimaging, etc. [1,2,3,4,5,6,7,8].

AIEgens exhibit remarkable advantages over traditional ACQ luminophores in aggregate and solid states, so constructing new AIEgens is of great importance for promoting the development of AIE research. Although enthusiastic efforts made by researchers have generated hundreds of AIEgens, many AIE cores contain cyclic or linear multiple vinyl groups in their structures. Ethylene derivatives may potentially encounter photo-oxidation and photobleaching problems. One effective strategy to improve stability is to construct AIEgens with sole aryl moieties. Meanwhile, the introduction of aromatic nitrogen heterocycles could help produce electron donor-acceptor structures to fine-tune the light emission in a wide range. Along these lines, tetraphenylpyrazine (TPP)-based AIEgens were designed based on the restriction of the intramolecular rotation mechanism and facilely synthesized under mild reaction conditions [9]. The TPP-based AIEgens exhibit remarkable advantages, such as facile preparation, efficient emission, good thermo-, photo- and chemostability [10,11].

**Chart 1:**
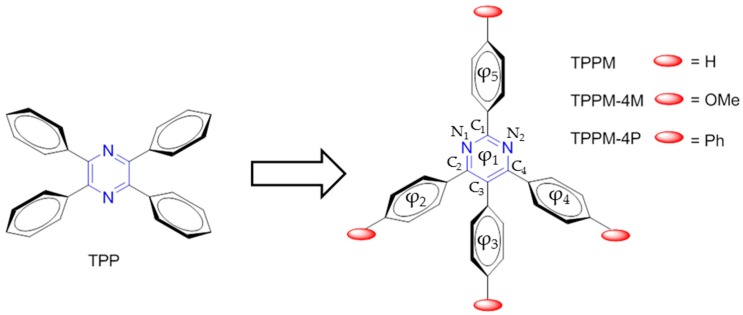
Molecular structures of AIEgens of tetraphenylpyrazine (TPP) and tetraphenylpyrimidine (TPPM) derivatives.

Inspired by the excellent performance of TPP derivatives, we designed and synthesized a new type of AIEgen based on the rarely explored tetraphenylpyrimidine (TPPM) (Chart 1). The central pyrimidine is anticipated to possess versatile functions such as protonation, hydrogen bond formation and chelation of the nitrogen atoms, which make the generated AIEgens applicable in developing advanced supramolecular assemblies and chemosensors [12]. Besides following the working mechanism of typical AIEgens, the TPPM undergoes intense high-frequency intramolecular bending motion in the pyrimidine ring, which contributes largely to the nonradiative decay channels. Accordingly, attaching electron-donating groups to the electron-withdrawing pyrimidine rings could extend the conjugation and rigidify the skeleton of the molecule, and suppress the high-frequency bending vibrations. As expected, the generated TPPM-4M and TPPM-4P attain higher luminescent efficiency in both solution and solid states than that of TPPM. Nanoaggregates based on TPPM derivatives show higher performance for detecting the picric acid with larger quenching constants than the typical AIEgen of tetraphenylethene (TPE). This is attributed to more efficient electron transfer processes originating from the formation of hydrogen bonds between pyrimidine rings of TPPM derivatives and the explosive molecules [13].

## 2. Results and Discussion

### 2.1. General Routes for Synthesis of TPPM Derivatives

**Scheme 1:**
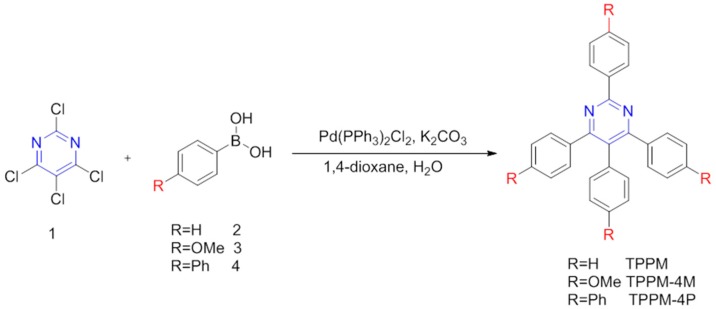
Synthetic routes to tetraphenylpyrimidine (TPPM) and its derivatives.

The general routes to synthesize TPPM and its derivatives are shown in Scheme 1. Commercially available starting materials of tetrachloropyrimidine (**1**) and aromatic boronic acids (**2**–**4**) could be readily converted to TPPM or its derivatives of TPPM-4M and TPPM-4P in satisfactory yields through Suzuki coupling [14]. Satisfactory data corresponding to the target TPPM derivatives were obtained from the ^1^H- and ^13^C-NMR, high-resolution mass measurements (Figures S1–S9). These compounds are constructed by aromatic moieties, which greatly enhance their thermal stability. The rapid drops on the thermal-gravity analysis (TGA) curves can be ascribed to the sublimation of samples (Figure S10). Therefore, we define the temperature of 5% loss of weight as *T_s_*_m_, measured to be 258 °C, 345 °C, and 466 °C for TPPM, TPPM-4M and TPPM-4P, respectively.

### 2.2. Photophysical Property of TPPM-Based AIEgens

**Figure 1:**
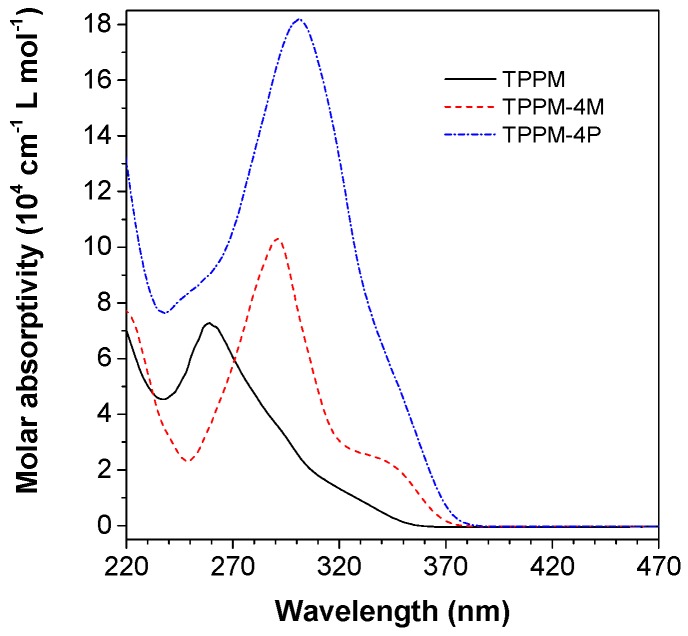
UV-vis absorption of TPPM and its derivatives in THF.

After confirming their structures, we investigated the photophysical properties of TPPM derivatives. We first measured their UV-vis absorption, shown in Figure 1. From TPPM to TPPM-4M and TPPM-4P, the maximum absorption peaks (*λ_abs_*) red shift from 259 to 301 nm because decorating TPPM with electron-donating methoxyl groups or additional phenyl rings readily extends the conjugation and facilitates the charge transfer from methoxyl groups or phenyl rings to the central pyrimidine core upon excitation. Meanwhile, the maximum molar absorbance is also improved owing to the extension of conjugation in TPPM-4M and TPPM-4P. These conclusions are supported by the theoretical calculation shown in Tables S1–S3. Furthermore, we measured the absorbance spectra in different solvents. The results show that the *λ_abs_* exhibit negligible shifts (Figures S11–S13).

**Figure 2:**
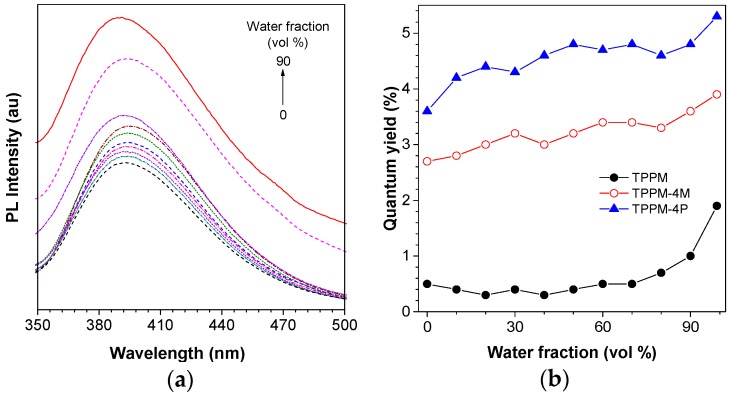
(**a**) PL spectra of TPPM in THF/water mixtures with different water fraction. Concentration: 10^−5^ M, *λ_ex_* = 300 nm; (**b**) Fluorescence quantum yields of TPPM and its derivatives vs. water fraction in THF/water mixtures.

Next, we investigated the photoluminescence (PL) behaviors of TPPM and its derivatives in the THF/water mixture with different water fraction (*f_w_*). Taking TPPM as an example (Figure 2a), its emission intensity enhances gradually with increasing *f_w_* from 0 to 70%. Afterwards, it rises markedly. The other derivatives show similar phenomena (Figures S14 and S15). These indicate that TPPM and its derivatives feature the unique aggregation-enhanced emission (AEE) characteristics. The fluorescence quantum yields (Φ_F_) measurement further confirmed their AEE feature. As shown in Figure 2b, the Φ_F_ values of three compounds increase in the THF/water mixtures with *f_w_* changing from 0 to 99%, although negligible fluctuation appears in the curves. Furthermore, the Φ_F_ values of TPPM, TPPM-4M and TPPM-4P powders are recorded to be 3.7%, 8.8% and 6.2%, respectively (Table S4). Interestingly, although negligible shift was observed in the absorbance spectra, the maximum emission peaks (*λ_em_*) of TPPM-4M and TPPM-4P exhibit an obvious bathochromic shift with an increase in the solvent polarity, while that of TPPM remains unchanged, which accounts for the enhanced charge transfer in TPPM derivatives by attaching the electron-donating groups.

### 2.3. Working Mechanism

**Figure 3:**
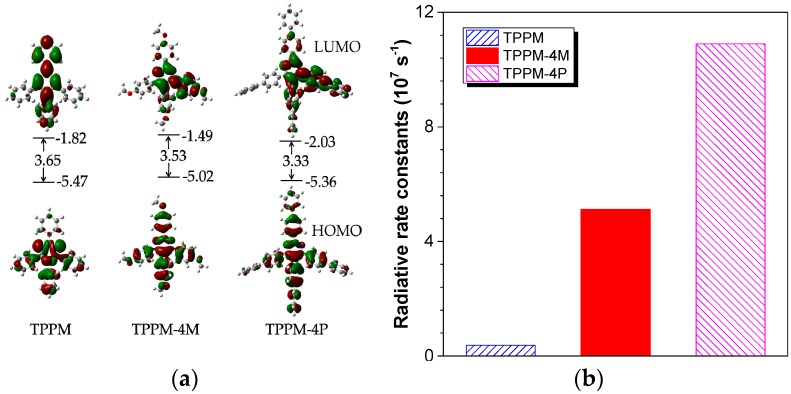
(**a**) Molecular orbital amplitude plots and energy levels of the HOMOs and the LUMOs of TPPM derivatives at the S_1_-geometry calculated at the B3LYP/6-31G(d, p) level; (**b**) Comparison of radiative rate constants of TPPM, TPPM-4M and TPPM-4P calculated in the isolated state.

We conducted a systematical theoretical investigation on the structure-property relationship of the TPPM derivatives aiming at illuminating the photo-physical mechanism. It is found that the HOMO of TPPM is mainly localized over the ϕ_2_, ϕ_3_, ϕ_4_ and the pyrimidine ring (Chart 1 and Figure 3a). After decorating with electron-donating methoxyl and phenyl groups, the charge density in HOMO is extended over these substituents and the energy increases in the order of TPPM, TPPM-4M and TPPM-4P. However, LUMO is mainly distributed on the ϕ_3_, ϕ_4_ and the central rings in both methoxyl and phenyl counterparts. The delocalization of LUMO increases in the order of TPPM-4M, TPPM and TPPM-4P, resulting in the decrease of energy in the same order. Overall, the energy gaps between HOMOs and LUMOs decrease in the order of TPPM, TPPM-4M and TPPM-4P. As expected, the calculated electric transition dipole moments increase from TPPM (1.17 Debye) to TPPM-4M (3.63 Debye) to TPPM-4P (5.54 Debye). As a consequence, the radiative rate constants of the three compounds increase in the order of TPPM, TPPM-4M and TPPM-4P (Figure 3b), which would contribute largely to the enhancement of the Φ_F_ of TPPM derivatives in solution after decorating with methoxyl and phenyl groups. These results are in good agreement with the measurement of Φ_F_ shown in Figure 2b.

The optimized molecular geometries of these three compounds are then examined and the data are listed in Table 1 and Table S5. It is obvious that the three compounds consist of a central stator and peripheral rotors, which are connected through single bonds. Relative to the central core, the peripheral phenyl rings ϕ_2_, ϕ_3_, and ϕ_4_ are hugely twisted due to the high steric congestion while the ϕ_5_ is almost planar without repulsion from adjacent groups at the S_0_ state. The highly twisted conformation of the TPPM derivatives could prevent from close packing in the condensed phase and reduce the possibility of forming emission quenching species. Upon excitation, the largest modifications appear in dihedral angles between ϕ_2_/ϕ_4_ and ϕ_1_ rings with the same decrease values of 13° in TPPM, keeping the approximate C2 symmetry. While the dihedral angles between ϕ_3_/ϕ_4_ and ϕ_1_ rings in the methoxyl and phenyl counterparts largely decrease with the values ranging from 11° to 20°, thus breaking the molecular C2 symmetry. The ϕ_5_ rings are still well conjugated with the central rings and the dihedral angles between ϕ_5_ and ϕ_1_ experience smallest variation upon excitation for all the three compounds. It is interesting that the bond angles in the pyrimidine ring experience intense modification upon excitation in TPPM, which has not been found in the reported AIEgens. The bond angles of central pyrimidine rings in three molecules are quite similar at S_0_ states. Nevertheless, the bond angles A(C_1_-N_1_-C_2_), A(C_1_-N_2_-C_4_), and A(N_1_-C_1_-N_2_) of TPPM change drastically with absolute values larger than 10° from S_0_ to S_1_ states, which would activate some non-radiative decay channels. All bond angles of pyrimidine rings of TPPM-4M and TPPM-4P show slight variation.

**Figure 4:**
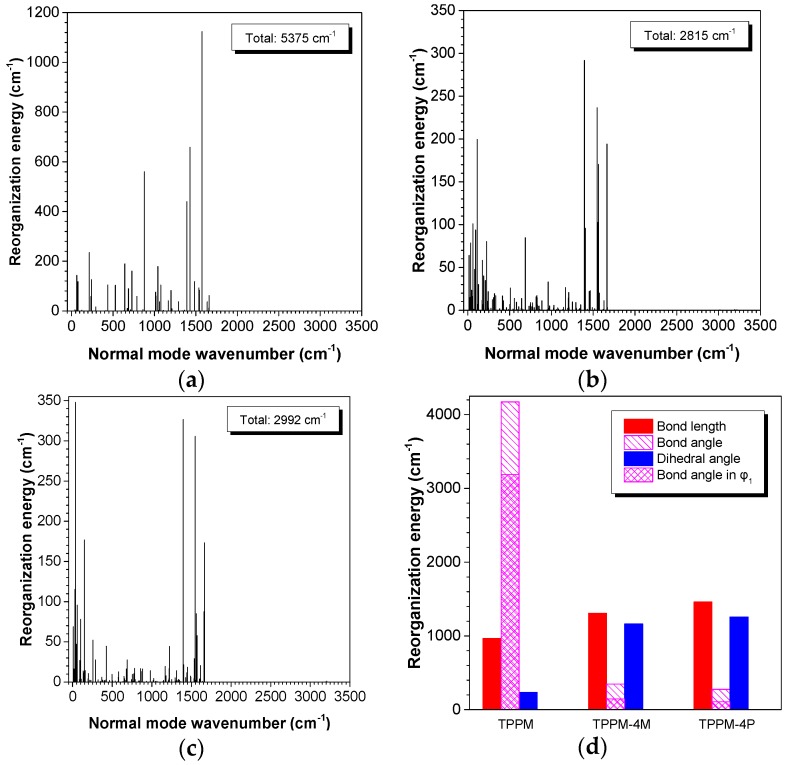
Presentation of the calculated reorganization energy versus the normal mode wavenumbers for (**a**) TPPM, (**b**) TPPM-4M, (**c**) TPPM-4P, and (**d**) contribution to the total reorganization energy from the bond length, the bond angle, the dihedral angle and the bond angle in the pyrimidine ring (ϕ_1_).

The reorganization energy in the S_1_/S_0_ geometrical relaxations characterizes the ability to produce internal conversion and provides essential details of the contribution from multiple intramolecular motions to the excited-state deactivation. Figure 4 illustrates the calculated reorganization energy versus the corresponding normal modes. It can be seen in TPPM that the high frequency modes contribute significantly to the total reorganization energy, which are predominantly ascribed to the stretching vibrations of C=C and C=N and the bending vibrations of bond angles in pyrimidine ring (Figure S16). Relative to that in TPPM, the total reorganization energy is found to decrease significantly, by about half in TPPM-4M and TPPM-4P, which implies the non-radiative decay would become much slower. Moreover, the contribution of bending motions turns weak and that of the low-frequency normal modes become dominant in TPPM-4M and TPPM-4P. The low-frequency modes are assigned as the rotational motions of the periphery phenyl rings as illustrated in Figures S17 and S18.

Further casting the reorganization energy onto the internal coordination depicts a clear picture of the specific structure-property relationship. As shown in Figure 4d, the reorganization energy from bond angles takes the largest proportion at 78% in TPPM, in which the bond angles in the pyrimidine core contributes as highly as 59% with the value of 3189 cm^−1^. This further confirms that the bending motions of the bond angles in the pyrimidine ring act as nonradiative decay channels and thus lead to low fluorescence quantum yields of TPPM in the dilute solution. Meanwhile, the contribution from bond angles to the reorganization energy are 12 and 9%, in which those from pyrimidine rings are 5 and 4% with values of 145 and 108 cm^−1^, in TPPM-4M and TPPM-4P, respectively. This indicates that attaching the methoxyl and phenyl groups to TPPM mitigates the bending motions of the central core by extending the conjugation degree and rigidifying the conformation of the whole molecule, thus improving the emission efficiency in both solution and aggregates.

**Figure 5:**
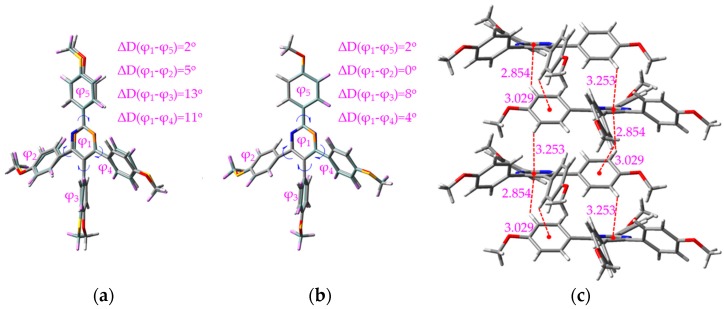
(**a**) Overlap diagram of optimized structures of S_0_ and S_1_ state of TPPM-4M in the gas phase and (**b**) in the crystal phase; (**c**) Single crystal packing diagram and intermolecular C–H‧‧‧π interactions (in angstrom) of TPPM-4M.

All three compounds present AEE phenomena as observed above. The light emission in the solid states of AIEgens is closely associated with the molecular packing modes [15,16,17]. We here take TPPM-4M as an example to figure out its mechanism and to establish the structure–property relationship. Figure 5 and Tables S6 and S7 provide the comparison of the molecular geometries at S_0_ and S_1_ states calculated in the gas and crystal phases, respectively. The calculated ground-state geometry in the solid phase is in good agreement with that in a single crystal, which indicates the rationality and reliability of the computational method adopted here. TPPM-4M adopts a twisted conformation with the dihedral angles between the peripheral phenyl rings and the central pyrimidine core ranging from 28 to 62°. Inspection of the single crystal packing manners shows that the highly twisted structure prevents molecules from close packing and multiple intermolecular C–H‧‧‧π interactions exist among the molecules. The C–H‧‧‧π interactions with the distances of 2.854 Ǻ between the ϕ_4_ ring and the pyrimidine ring and of 3.029 Ǻ between ϕ_3_ and ϕ_4_ generate effective constraint, and then the variation of dihedral angles of D(ϕ_1_–ϕ_2_), D(ϕ_1_–ϕ_3_) and D(ϕ_1_–ϕ_4_) are as expected to be reduced to 0°, 8° and 4°, respectively (Figure 5a,b). Hence, TPPM-4M in solid phase experiences less change compared with those in the gas phase, which would correspondingly cripple the non-radiative deactivation in the form of heat.

**Figure 6:**
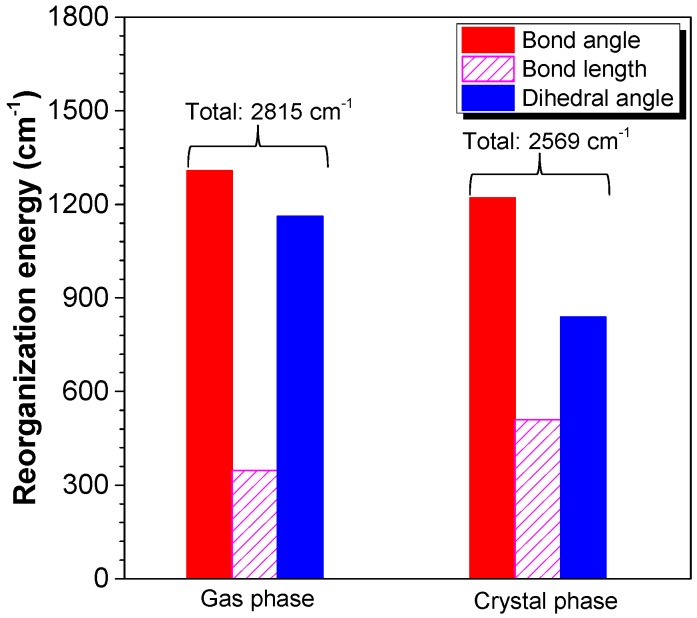
Contribution to the total reorganization energy in the gas and the crystal phase from the bond length, the bond angle, and the dihedral angle.

The features of the geometrical modification are sure to produce different properties from the gas to the solid phase. It is most obvious that the total reorganization energy is reduced from 2815 in the gas phase to 2569 cm^−1^ in the crystal phase for TPPM-4M. The reorganization energy from bond lengths and bond angles in the gas phase almost equals to that in the crystal phase due to the similar variation pattern of these structural parameters. However, the reorganization energy from dihedral angles decreases from 1162 to 839 cm^−1^ (Figure 6), indicating the low-frequency rotational motion of peripheral phenyl groups is blocked. Hence, the calculated nonradiative decay rate constants decline sharply by five orders of magnitude in the condensed state, compared to the isolated state. The radiative rate constant of the condensed state is in the same magnitude as that of the isolated one because their electric transition dipole moments are close (Table 2). These evidently reveal the restriction of the non-radiative decay pathways and the enhancement of light emission in the aggregate state.

### 2.4. Application in Explosive Detection

**Figure 7:**
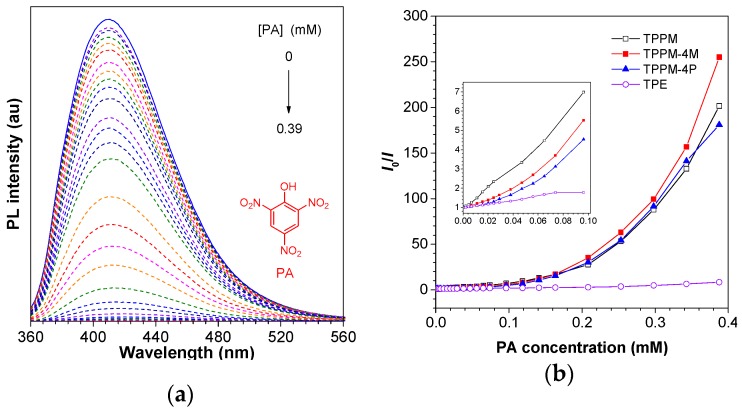
(**a**) PL spectra of TPPM-4M with addition of different amounts of PA in THF/water mixture (*f_w_* = 99%). Concentration of TPPM-4M = 10^−5^ M. Excitation wavelength = 340 nm. (**b**) Stern-Volmer plots of *I*_0_/*I* versus [PA] in THF/water mixture (*f_w_* = 99%). *I* = peak intensity. *I*_0_ = peak intensity at [PA] = 0 mM.

Nanoaggregates of TPPM and its derivatives can serve as efficient fluorescent probes for explosive detection owning to their three-dimensional topological structures, which can generate numerous internal voids for interacting with analytes and migration pathways for excitons [18,19,20]. Herein, we chose the commercially available picric acid (PA) as a model explosive to monitor the variation of PL intensity of aggregates of TPPM derivatives in THF/water mixture (*f_w_* = 99%). As shown in Figure 7a, the PL of TPPM-4M is progressively quenched with increasing PA concentration ([PA]). Obvious light annihilation was observed even at a [PA] as low as 0.5 ppm. At a [PA] of 0.39 mM, the emission is completely extinguished. The PL quenching spectra of the other two compounds are given in Figures S20–S22. Upward-bending variation curves were obtained in the Stern-Volmer plots of PL intensity ratio (*I*_0_/*I*) versus the PA concentration instead of straight lines. The quenching constants of 58,734, 41,703 and 32,269 M^−1^ of TPPM, TPPM-4M and TPPM-4P are deduced from the plots when PA concentration is below 0.1 mM, respectively, which are much higher than that of TPE of 9299 M^−1^ (Inset in Figure 7b). The upward-bending curves in the Stern-Volmer plots after 0.1 mM show the super-amplification quenching effect.

**Figure 8:**
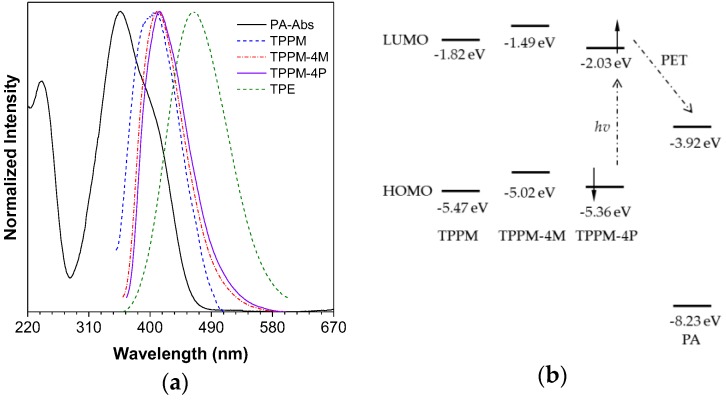
(**a**) Normalized UV-Vis absorption spectra of PA and PL spectra of TPPM, TPPM-4M, TPPM-4P and TPE in THF/water mixture (*f_w_* = 99%); (**b**) Calculated energy level diagrams of TPPM derivatives and the PA.

The fluorescence quenching could be generally ascribed to (i) the resonance energy transfer from the excited state of probe molecules to the ground state of analytes due to spectra overlap between emission bands of the former and absorption bands of the latter, and (ii) the photo-induced electron transfer (PET) from the probe to PA [21,22,23,24]. As can be seen in Figure 8a, all the emission bands of TPPM derivatives and TPE overlap with the absorption bands of PA, indicating that the resonance energy transfer could contribute largely to the PL quenching process. However, the more remarkable sensitivity of TPPM derivatives to PA, compared to TPE, is found to originate from the hydrogen-bond facilitated electron transfer process. The calculated relative positions between the probe and the PA are shown in Figure S23. The pyrimidine rings are able to form N‧‧‧H hydrogen bonds with PA molecules with distances ranging from 1.666 to 2.058 Ǻ. The lower complexation energy between TPPM derivatives and the PA molecule than TPE reveals higher stability of such complexation patterns through hydrogen bonds between TPPM derivatives and the PA molecule (Table S8). The N‧‧‧H hydrogen bonds can shorten the distance between fluorophores and PA molecules and facilitate electron transfer driven by the LUMO-LUMO offsets from probes to PA molecules (Figure 8b). Hence, TPPM derivatives attain larger quenching constants than TPE and show higher sensing performance for detecting explosives.

## 3. Materials and Methods

### 3.1. Synthesis of TPPM

Tetrachloropyrimidine **1** (0.22 g, 1 mmol), phenylboronic acid **2** (0.54 g, 4.4 mmol), Pd(PPh_3_)_2_Cl_2_ (0.035 g, 0.05 mmol), and K_2_CO_3_ (0.70 g, 5 mmol) were added to an oven-dried round bottom flask. The flask was filled with nitrogen. The 1,4-dioxane (7.5 mL) and distilled water (2.5 mL) were injected by syringe. The reaction mixture was stirred at 150 °C for 10 h. After cooling to room temperature, the products were extracted with dichloromethane (3 × 25 mL), and the organic layer was washed with water, dried over magnesium sulfate and filtration. Then the TPPM was isolated in a 81.6% yield (0.31 g) as a white solid through the silica-gel chromatographic column separation using a mixture of petroleum ether/dichloromethane as eluent and rotary evaporation under reduced pressure.

^1^H-NMR (500 MHz, DMSO-*d*_6_): δ (TMS, ppm) 8.57–8.47 (m, 2H), 7.64–7.51 (m, 3H), 7.42–7.16 (m, 13H), 7.12–7.04 (m, 2H). ^13^C-NMR (125 MHz, DMSO-*d*_6_): δ (TMS, ppm) 165.65, 162.15, 138.90, 137.55, 136.48, 131.38, 131.31, 129.97, 129.72, 129.20, 129.16, 128.62, 128.35, 128.21, 127.93. HRMS (MALDI-TOF): *m*/*z* 385.1700 ([M + H]^+^). Calcd for C_28_H_21_N_2_ 385.1705.

### 3.2. Synthesis of TPPM-4M

Tetrachloropyrimidine **1** (0.22 g, 1 mmol), 4-methoxyl-phenylboronic acid **3** (0.67 g, 4.4 mmol), Pd(PPh_3_)_2_Cl_2_ (0.035 g, 0.05 mmol), and K_2_CO_3_ (0.70 g, 5 mmol) were added to an oven-dried round bottom flask. The flask was filled with nitrogen. The 1,4-dioxane (7.5 mL) and distilled water (2.5 mL) were injected by syringe. The reaction mixture was stirred at 150 °C for 10 h. After cooling to room temperature, the products were extracted with dichloromethane (3 × 50 mL), and the organic layer was washed with water, dried over magnesium sulfate and filtration. Then the TPPM-4M was isolated in a 78.0% yield (0.39 g) as a white solid through the silica-gel chromatographic column separation using dichloromethane as eluent and rotary evaporation under reduced pressure.

^1^H-NMR (500 MHz, DMSO-*d*_6_): δ (TMS, ppm) 8.47–8.40 (m, 2H), 7.34–7.28 (m, 4H), 7.13–7.05 (m, 2H), 7.00–6.90 (m, 2H), 6.89–6.75 (m, 6H), 3.85 (s, 3H), 3.75 (s, 6H), 3.72 (s, 3H). ^13^C-NMR (125 MHz, DMSO-*d*_6_): δ (TMS, ppm) 164.89, 161.91, 161.58, 159.96, 158.77, 132.42, 131.56, 131.45, 130.34, 129.88, 127.71, 114.46, 114.39, 113.63, 55.78, 55.61, 55.45. HRMS (MALDI-TOF): *m*/*z* 504.2036 ([M]^+^). Calcd for C_32_H_28_N_2_O_4_ 504.2049.

### 3.3. Synthesis of TPPM-4P

Tetrachloropyrimidine **1** (0.22 g, 1 mmol), biphenylboronic acid **4** (0.87 g, 4.4 mmol), Pd(PPh_3_)_2_Cl_2_ (0.035 g, 0.05 mmol), and K_2_CO_3_ (0.70 g, 5 mmol) were added to an oven-dried round bottom flask. The flask was filled with nitrogen. The 1,4-dioxane (7.5 mL) and distilled water (2.5 mL) were injected by syringe. The reaction mixture was stirred at 150 °C for 10 h. After cooling to room temperature, the products were extracted with dichloromethane (3 × 25 mL), and the organic layer was washed with water, dried over magnesium sulfate and filtration. Then the TPPM-4P was isolated in a 72.5% yield (0.50 g) as a white solid through the silica-gel chromatographic column separation using a mixture of petroleum ether/dichloromethane and rotary evaporation under reduced pressure.

^1^H-NMR (500 MHz, CD_2_Cl_2_): δ (TMS, ppm) 8.76 (d, *J* = 8.5 Hz, 2H), 7.80 (d, *J* = 8.5 Hz, 2H), 7.76–7.71 (m, 2H), 7.65–7.29 (m, 28H), 7.21 (d, *J* = 8.3 Hz, 2H). ^13^C-NMR (125 MHz, CD_2_Cl_2_): δ (TMS, ppm) 166.42, 163.68, 144.58, 142.65, 141.79, 141.51, 141.39, 141.26, 139.08, 137.97, 137.00, 132.89, 131.82, 130.16, 130.15, 130.11, 130.08, 130.06, 129.03, 128.94, 128.82, 128.45, 128.40, 128.30, 128.21, 128.11, 127.67. HRMS (MALDI-TOF): *m*/*z* 689.2876 ([M]^+^). Calcd for C_52_H_36_N_2_ 689.2957.

### 3.4. Methods for Theoretical Calculation

All the geometrical optimizations and frequency calculations were carried out using the (TD)B3LYP/6-31G(d, p) at the S_0_ (S_1_) for the studied compounds in the gas phase in the Gaussian 09 package [25]. The corresponding calculations for them in the solid phase were performed using the combined quantum chemistry and molecular chemistry (QM/MM) approach. The QM/MM model was set up by cutting a cluster containing 64 TPPM-4M molecules from the single crystal structure, the central molecule was treated as the QM part and the surrounding ones acted as the MM part. (Figure S19). The QM/MM calculation was performed by using the Chemshell 3.5 package [26], interfacing the Turbomole 6.5 [27] for QM at the B3LYP/6-31G(d) level and the DL_POLY [28] for MM with the general Amber force field (GAFF) [29]. The analytical frequencies for the S_0_ state at the DFT level and numerical frequencies for the S_1_ state at the TD-DFT level. The calculation of reorganization energy, radiative and nonradiative rate constants were carried out in the MOMAP package developed by Shuai group [30,31,32,33,34,35]. The formation of hydrogen bonds was evaluated by optimizing the relative position between the sensor molecule and the PA molecule at the ωB97XD/6-31G(d, p) level in the Gaussian 09 package.

## 4. Conclusions

A new type of AIEgen based on tetraphenylpyrimidine (TPPM) was rationally designed and facilely prepared. Theoretical investigation indicates that high-frequency bending motions of bond angles in the pyrimidine ring dominate the nonradiative decay process in TPPM. Attaching electron-donating methoxyl and phenyl groups to TPPM extends the conjugation and rigidifies the molecule skeleton and thus mitigates bending motion of the central core. As a result, relative to the TPPM, the reorganization energies of TPPM-4M and TPPM-4P are decreased sharply by more than half, greatly constraining the nonradiative decay. Meanwhile, the electric transition dipole moments of TPPM-4M and TPPM-4P are greatly enhanced, inducing faster radiative decay. Thus, the emission efficiency is promoted in both the solution and the solid state. Furthermore, multiple intermolecular interaction and steric hindrance existing in the aggregates block the low-frequency rotational motions of peripheral rotors and thus intensify the solid-state emission of TPPM derivatives. TPPM derivatives attain higher performance in sensing PA than the typical TPE, which is ascribed to the electron transfer process facilitated by the formation of hydrogen bonds between the pyrimidine and PA. Hence, nanoaggregates based on TPPM derivatives are fairly applicable in practical detection of explosive dissolved in water. Thus, these TPPM derivatives not only enrich the family of AIEgens but could also potentially be applied in optoelectronic and biological areas.

## Figures and Tables

**Table 1:** Selected dihedral angles and bond angles (in degree) for isolated TPPM, TPPM-4M, TPPM-4P molecules in the S_0_ and the S_1_ state.

	TPPM			TPPM-4M			TPPM-4P		
	S_0_	S_1_	Δ(S_1_ − S_0_)	S_0_	S_1_	Δ(S_1_ − S_0_)	S_0_	S_1_	Δ(S_1_ − S_0_)
**D(ϕ_1_–ϕ_2_)**	40	27	−13	37	42	5	40	47	7
**D(ϕ_1_–ϕ_3_)**	63	65	2	62	49	−13	62	42	−20
**D(ϕ_1_–ϕ_4_)**	40	27	−13	37	26	−11	40	25	−15
**D(ϕ_1_–ϕ_5_)**	4	7	3	5	7	2	4	7	3
**A(C_1_-N_1_-C_2_)**	118	128	10	118	117	−1	118	117	−1
**A(C_1_-N_2_-C_4_)**	118	128	10	118	121	3	118	121	3
**A(N_1_-C_2_-C_3_)**	122	121	−1	122	123	1	122	123	1
**A(N_2_-C_4_-C_3_)**	122	121	−1	121	118	−3	122	118	−4
**A(N_1_-C_1_-N_2_)**	125	111	−14	125	125	0	125	125	0
**A(C_2_-C_3_-C_4_)**	116	112	−4	116	116	0	116	116	0

**Table 2:** The calculated electric transition dipole moments (*µ*), radiative rate constants (*k_r_*) and nonradiative rate constants (*k_ic_*) of TPPM-4M in the gas phase and the crystal phase (*T* = 300 K).

300 K	*µ* (Debye)	*k_r_* (s^−1^)	*k_ic_* (s^−1^)
Gas	3.63	5.12 × 10^7^	3.30 × 10^11^
Crystal	3.21	3.54 × 10^7^	2.34 × 10^6^

## Supplementary Materials

The supplementary materials are available online.
