# Supplementary material for: Tetraphenylpyrimidine-Based AIEgens: Facile Preparation, Theoretical Investigation and Practical Application
Source: Molecules. 2017 Oct 10;22(10):1679. doi: 10.3390/molecules22101679 (PMC6151576; doi:10.3390/molecules22101679)
Supplement: Supplementary file 1 [file molecules-22-01679-s001.docx]

**Electronic Supplementary Materials**

Tetraphenylpyrimidine-based AIEgens: Facile Preparation, Theoretical Investigation and Practical Application

Junkai Liu ^1,2^, Lingxiang Pan ^1^, Qian Peng ^2,^* and Anjun Qin ^1,^*

^1^ State Key Laboratory of Luminescent Materials and Devices, South China University of Technology, Guangzhou 510640, China; [ipowerjkl@outlook.com](mailto:ipowerjkl@outlook.com) (J.L.); [panlx2014@hotmail.com](mailto:panlx2014@hotmail.com) (L.P.);

^2^ Key Laboratory of Organic solids, Beijing National Laboratory for Molecular Sciences, Institute of Chemistry, Chinese Academy of Sciences, Beijing 100190, China;

* Correspondence: [qpeng@iccas.ac.cn](mailto:qpeng@iccas.ac.cn); Tel.: +86-10-82616830; msqinaj@scut.edu.cn; Tel.: +86 -20-22237065

**Table of Contents**

[**Figure S1.** ^1^H NMR spectrum of TPPM in DMSO-*d_6_*. The solvent peaks are marked with asterisk. S4](#_Toc492594514)

[**Figure S2.** ^13^C NMR spectrum of TPPM in DMSO-*d_6_*. The solvent peak is marked with asterisk. S4](#_Toc492594515)

[**Figure S3.** ^1^H NMR spectrum of TPPM-4M in DMSO-*d_6_*. The solvent peaks are marked with asterisks. S5](#_Toc492594516)

[**Figure S4.** ^13^C NMR spectrum of TPPM-4M in DMSO-*d_6_*. The solvent peak is marked with asterisk. S5](#_Toc492594517)

[**Figure S5.** ^1^H NMR spectrum of TPPM-4P in CD_2_Cl_2_. The solvent peak is marked with asterisk. S6](#_Toc492594518)

[**Figure S6.** ^13^C NMR spectrum of TPPM-4P in CD_2_Cl_2_. The solvent peak is marked with asterisk. S6](#_Toc492594519)

[**Figure S7.** HRMS spectrum of TPPM. S7](#_Toc492594520)

[**Figure S8.** HRMS spectrum of TPPM-4M. S7](#_Toc492594521)

[**Figure S9.** HRMS spectrum of TPPM-4P. S8](#_Toc492594522)

[**Figure S10.** TGA curves of TPPM and its derivatives under nitrogen at a heating rate of 10 ^o^C/min. S13](#_Toc492594531)

[**Figure S11.** (a) UV-vis absorption and (b) PL spectra of TPPM in different solvent. S14](#_Toc492594532)

[**Figure S12.** (a) UV-vis absorption and (b) PL spectra of TPPM-4M in different solvent. S14](#_Toc492594533)

[**Figure S13.** (a) UV-vis absorption and (b) PL spectra of TPPM-4P in different solvent. S15](#_Toc492594534)

[**Figure S14.** PL spectrum of TPPM-4M in THF/water mixtures with different water fraction. Concentration: 10^-5^ M, *λ_ex_* = 340 nm. S16](#_Toc492594535)

[**Figure S15.** PL spectrum of TPPM-4P in THF/water mixtures with different water fraction. Concentration: 10^-5^ M, *λ_ex_* = 345 nm. S16](#_Toc492594536)

[**Table S1**. The calculated natural transition orbitals of the absorption process of TPPM. S17](#_Toc492594537)

[**Table S2**. The calculated natural transition orbitals of the absorption process of TPPM-4M. S18](#_Toc492594538)

[**Table S3**. The calculated natural transition orbitals of the absorption process of TPPM-4P. S19](#_Toc492594539)

[**Table S4.** Optical and thermal properties of TPPM and its derivatives. S21](#_Toc492594540)

[**Table S5.** Selected bond lengths (in angstrom) of TPPM and its derivatives. S22](#_Toc492594541)

[**Figure S16.** Diagrammatic illustration of selected normal modes with reorganization energy (*λ*) larger than 100 cm^-1^ of TPPM in the gas phase. S22](#_Toc492594542)

[**Figure S17.** Diagrammatic illustration of selected normal modes with reorganization energy (*λ*) larger than 50 cm^-1^ of TPPM-4M in the gas phase. S23](#_Toc492594543)

[**Figure S18.** Diagrammatic illustration of selected normal modes with reorganization energy (*λ*) larger than 50 cm^-1^ of TPPM-4P in the gas phase. S24](#_Toc492594544)

[**Figure S19.** Model cluster cut from the single crystal structure for QM/MM calculation. S25](#_Toc492594545)

[**Table S6.** Selected bond lengths (in angstrom) of TPPM-4M in the single crystal and corresponding parameters calculated in the gas and the crystal phase. S25](#_Toc492594546)

[**Table S7.** Selected bond angles (in degree) of TPPM-4M in the single crystal and corresponding parameters calculated in the gas and the crystal phase. S25](#_Toc492594547)

[**Figure S20.** PL spectrum of TPPM with addition of different amounts of PA in THF/water mixture (*f_w_* = 99%). Concentration of TPPM = 10^-5^ M. Excitation wavelength = 300 nm. S26](#_Toc492594548)

[**Figure S21.** PL spectrum of TPPM-4P with addition of different amounts of PA in THF/water mixture (*f_w_* = 99%). Concentration of TPPM-4P = 10^-5^ M. Excitation wavelength = 345 nm. S26](#_Toc492594549)

[**Figure S22.** PL spectrum of TPE with addition of different amounts of PA in THF/water mixture (*f_w_* = 99%). Concentration of TPE = 10^-5^ M. Excitation wavelength = 309 nm. S27](#_Toc492594550)

[**Figure S23.** Hydrogen bonds formed among TPPM derivatives and PA molecules calculated through conformation optimization at ωb97XD/6-31G(d, p) level. S27](#_Toc492594551)

[**Table S8.** Calculated lengths of hydrogen bonds and complexation energy of TPPM derivatives and PA. S28](#_Toc492594552)


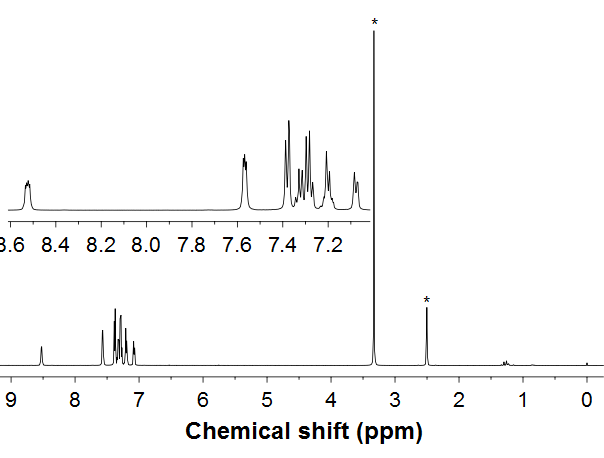


**Figure S1.** ^1^H NMR spectrum of TPPM in DMSO-*d_6_*. The solvent peaks are marked with asterisk.


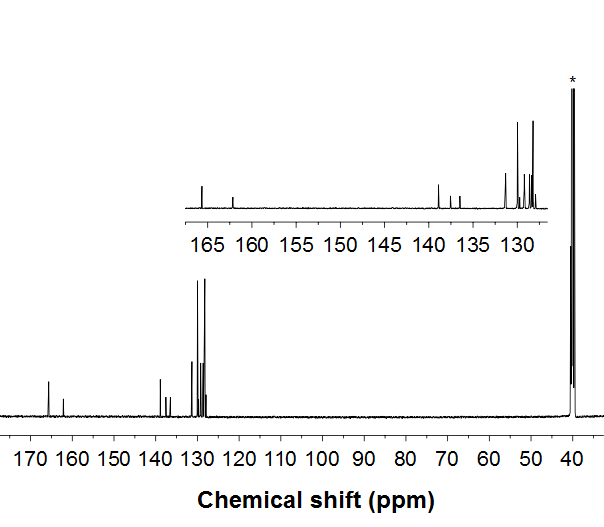


**Figure S2.** ^13^C NMR spectrum of TPPM in DMSO-*d_6_*. The solvent peak is marked with asterisk.


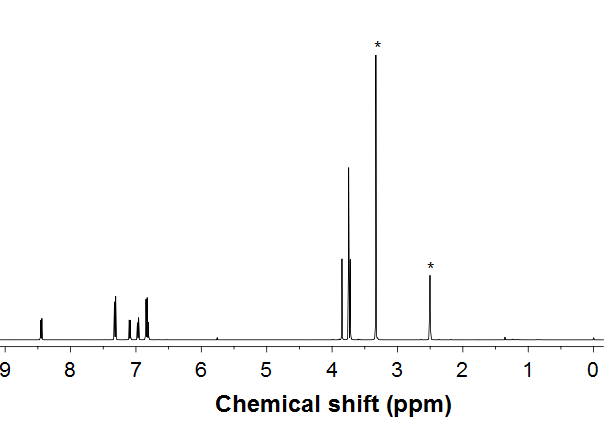


**Figure S3.** ^1^H NMR spectrum of TPPM-4M in DMSO-*d_6_*. The solvent peaks are marked with asterisks.


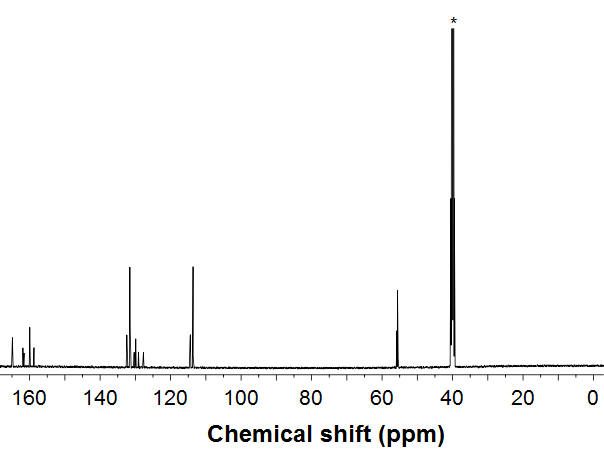


**Figure S4.** ^13^C NMR spectrum of TPPM-4M in DMSO-*d_6_*. The solvent peak is marked with asterisk.


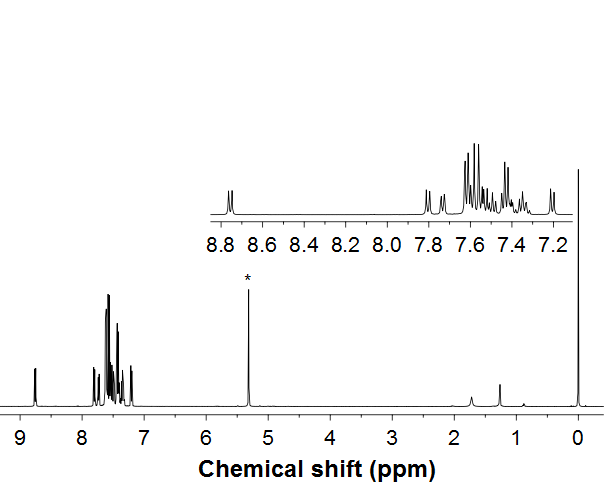


**Figure S5.** ^1^H NMR spectrum of TPPM-4P in CD_2_Cl_2_. The solvent peak is marked with asterisk.


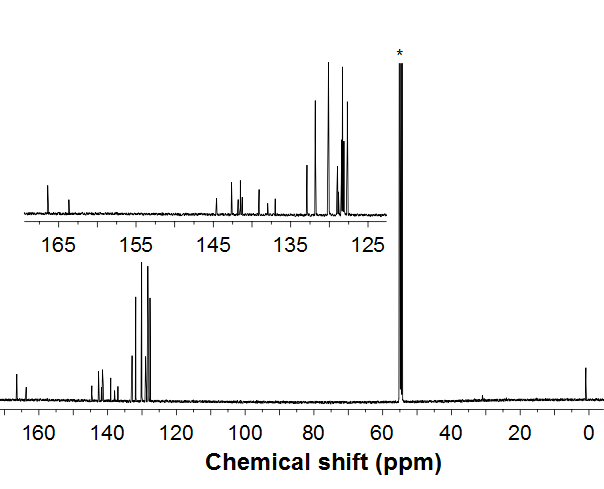


**Figure S6.** ^13^C NMR spectrum of TPPM-4P in CD_2_Cl_2_. The solvent peak is marked with asterisk.

**Figure S7.** HRMS spectrum of TPPM.

**Figure S8.** HRMS spectrum of TPPM-4M.

**Figure S9.** HRMS spectrum of TPPM-4P.

**Figure S10.** TGA curves of TPPM and its derivatives under nitrogen at a heating rate of 10 ^o^C/min.

.

|   (a) |   (b) |
| --- | --- |

**Figure S11.** (a) UV-vis absorption and (b) PL spectra of TPPM in different solvent.

|   (a) |   (b) |
| --- | --- |

**Figure S12.** (a) UV-vis absorption and (b) PL spectra of TPPM-4M in different solvent.

|   (a) |   (b) |
| --- | --- |

**Figure S13.** (a) UV-vis absorption and (b) PL spectra of TPPM-4P in different solvent.

**Figure S14.** PL spectrum of TPPM-4M in THF/water mixtures with different water fraction. Concentration: 10^-5^ M, *λ_ex_* = 340 nm.

**Figure S15.** PL spectrum of TPPM-4P in THF/water mixtures with different water fraction. Concentration: 10^-5^ M, *λ_ex_* = 345 nm.

**Table S1**. The calculated natural transition orbitals of the absorption process of TPPM.

| TPPM | Hole |  | Electron |
| --- | --- | --- | --- |
| S_0_–S_1_ | 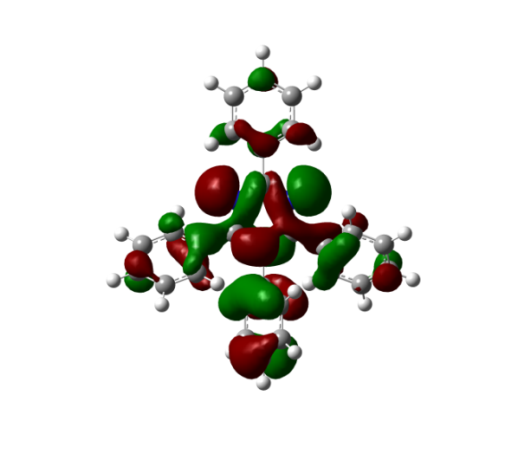 | 3.75 eV / 331 nm  *f* = 0.0674 | 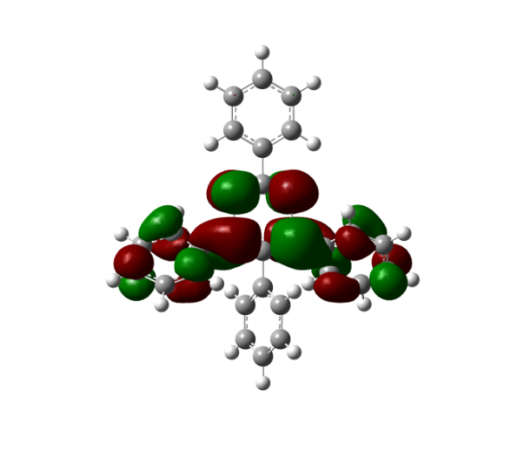 |
| S_0_–S_2_ | 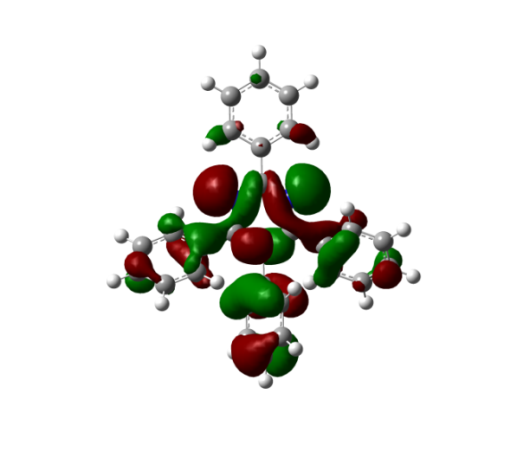 | 3.85 eV / 322 nm  *f* = 0.0586 | 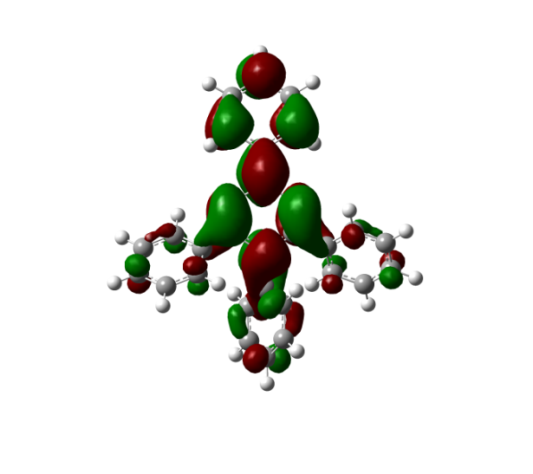 |
| S_0_–S_3_ | 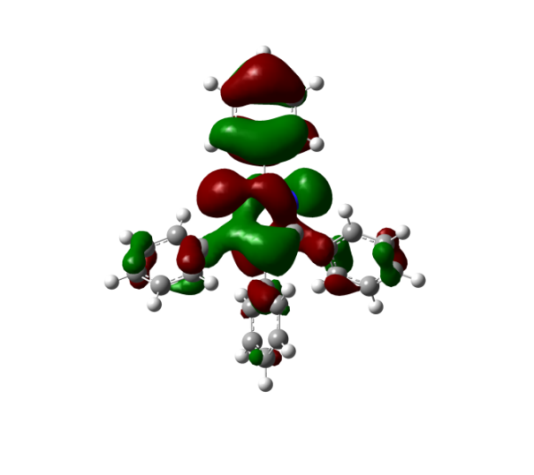 | 4.08 eV / 304 nm  *f* = 0.0511 | 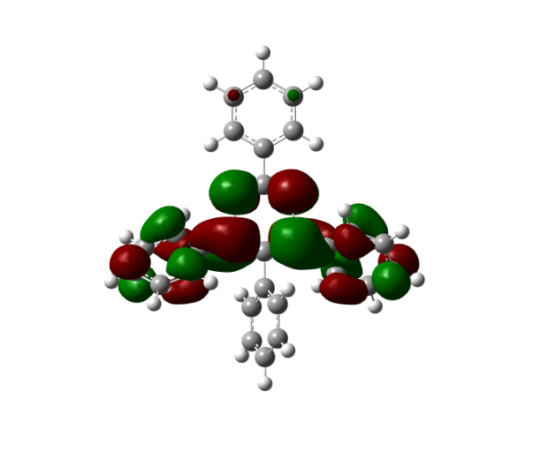 |
| S_0_–S_4_ | 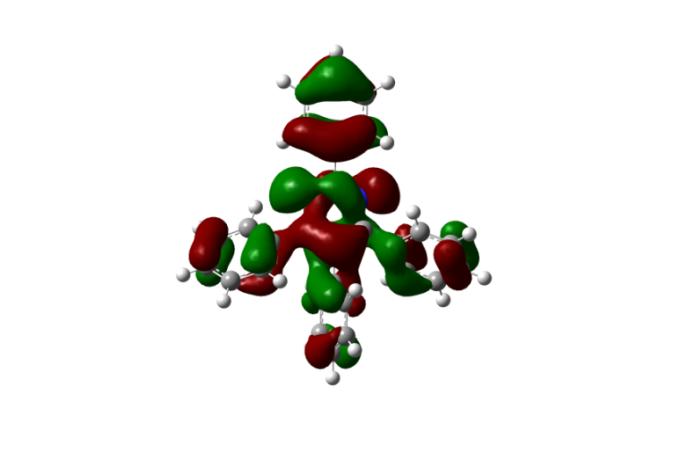 | 4.30 eV / 288 nm  *f* = 0.3387 | 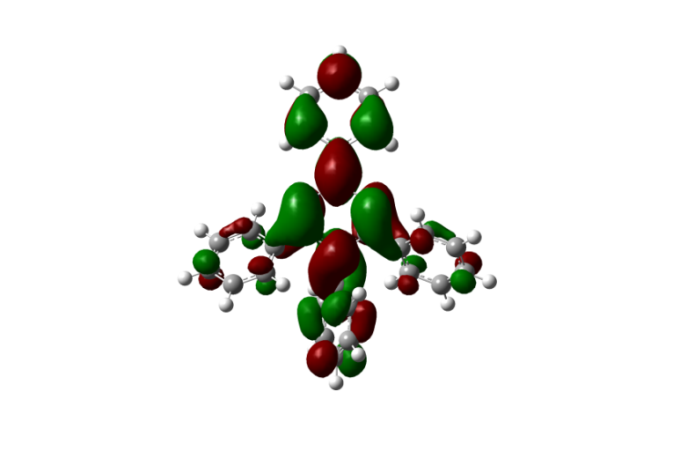 |
| S_0_–S_5_ | 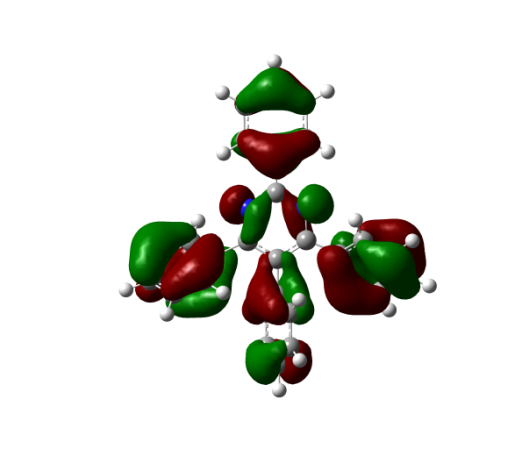 | 4.52 eV / 274 nm  *f* = 0.1000 | 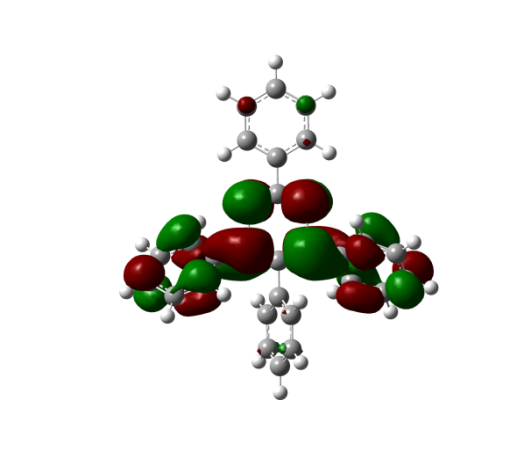 |

**Table S2**. The calculated natural transition orbitals of the absorption process of TPPM-4M.

| TPPM-4M | Hole |  | Electron |
| --- | --- | --- | --- |
| S_0_–S_1_ | 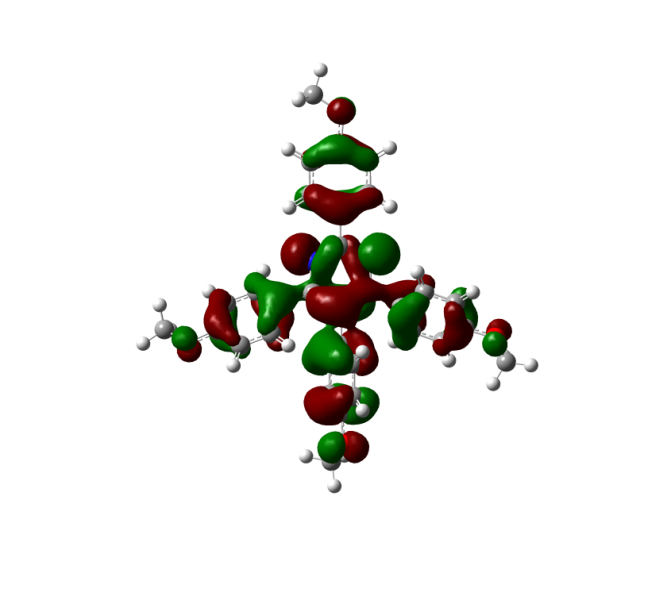 | 3.55eV / 349 nm  *f* = 0.1503 | 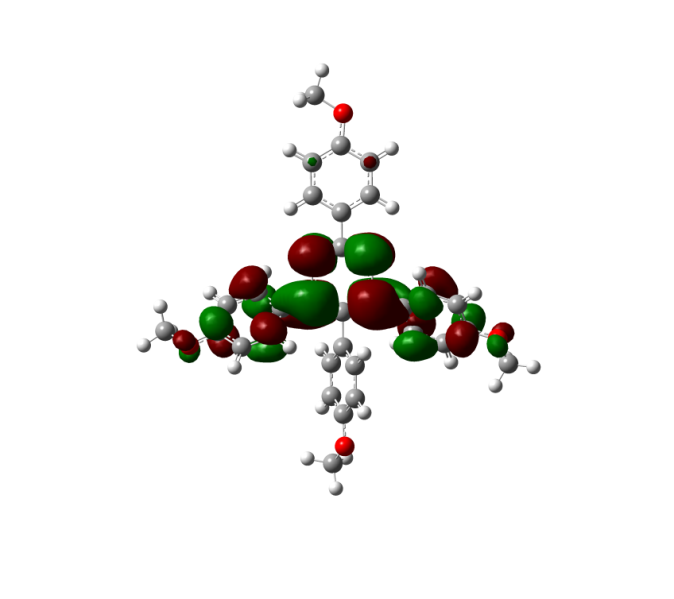 |
| S_0_–S_2_ | 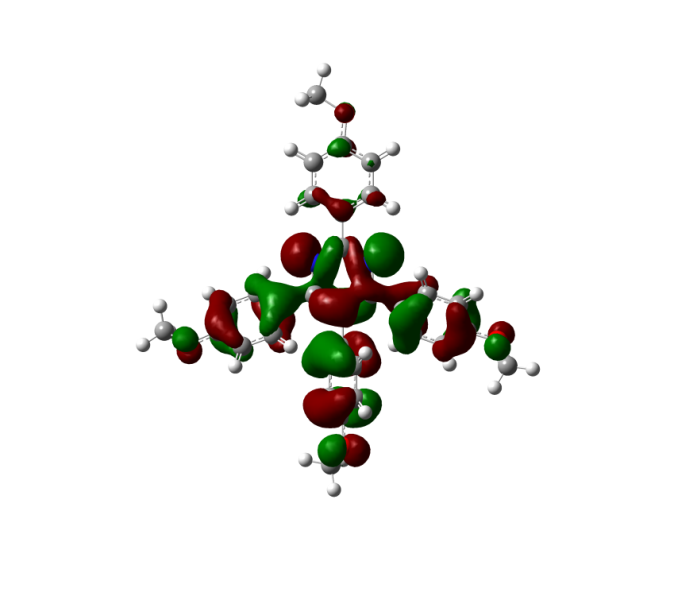 | 3.71 eV / 335 nm  *f* = 0.1574 | 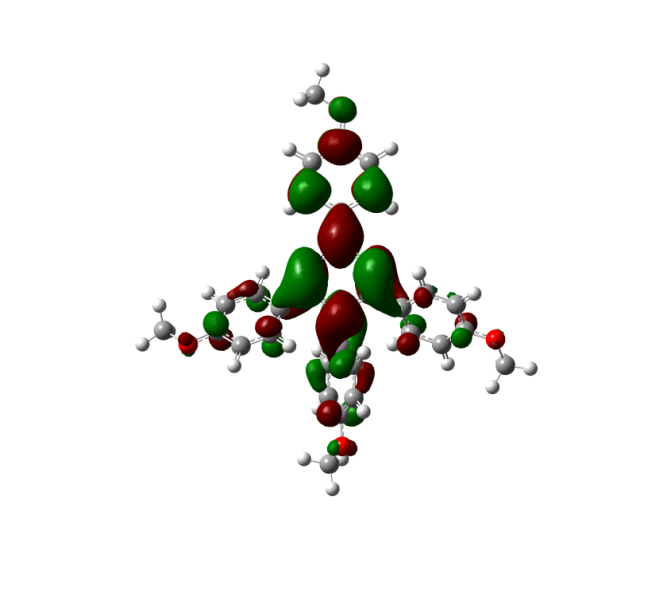 |
| S_0_–S_4_ | 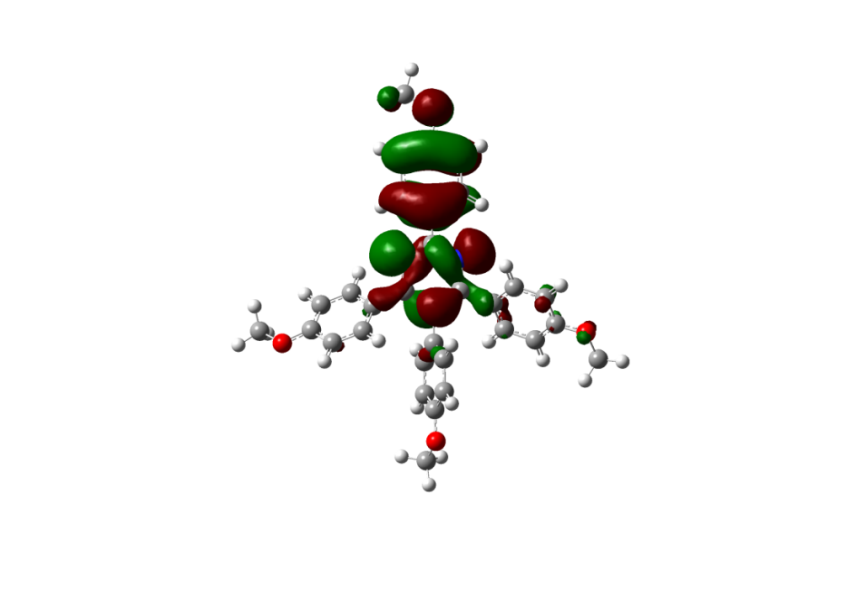 | 4.12 eV / 301 nm  *f* = 0.5934 | 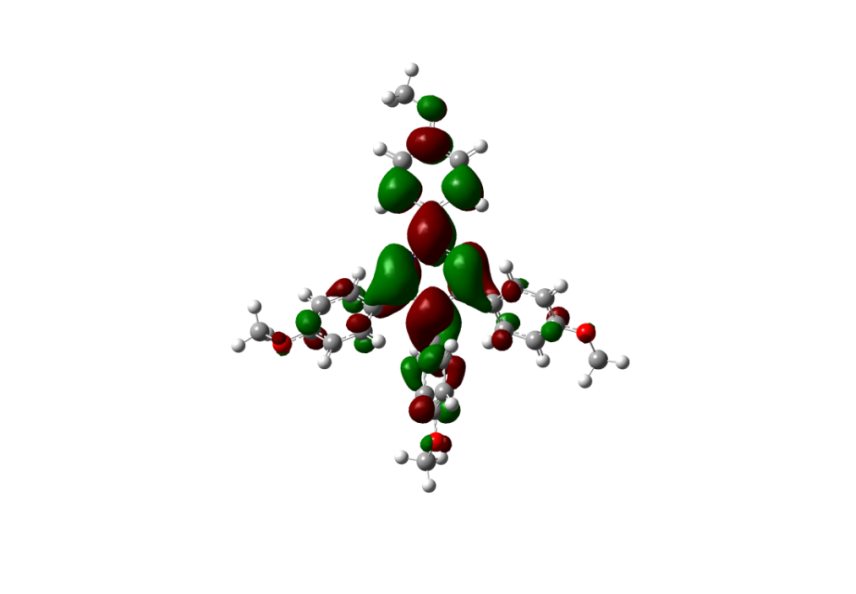 |
| S_0_–S_6_ | 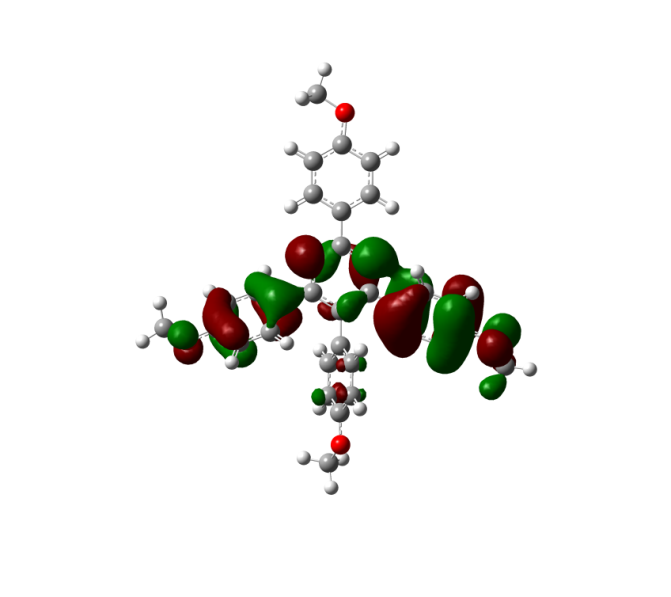 | 4.21 eV / 294 nm  *f* = 0.3687 | 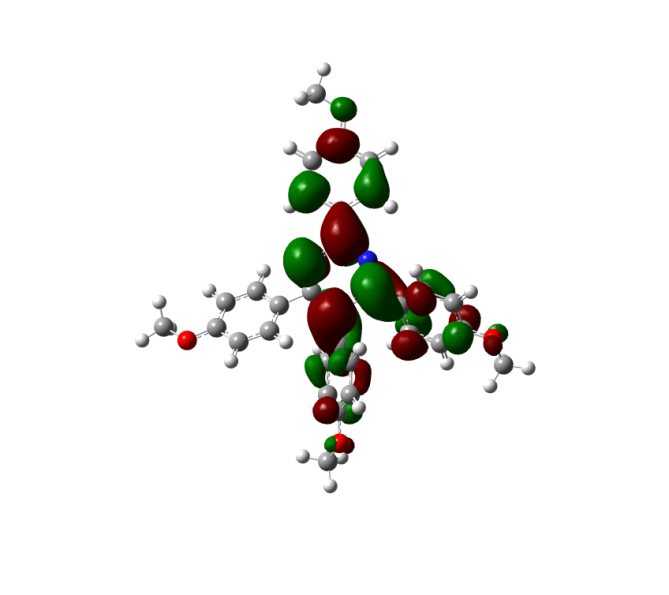 |

**Table S3**. The calculated natural transition orbitals of the absorption process of TPPM-4P.

| TPPM-4P | Hole |  | Electron |
| --- | --- | --- | --- |
| S_0_–S_1_ | 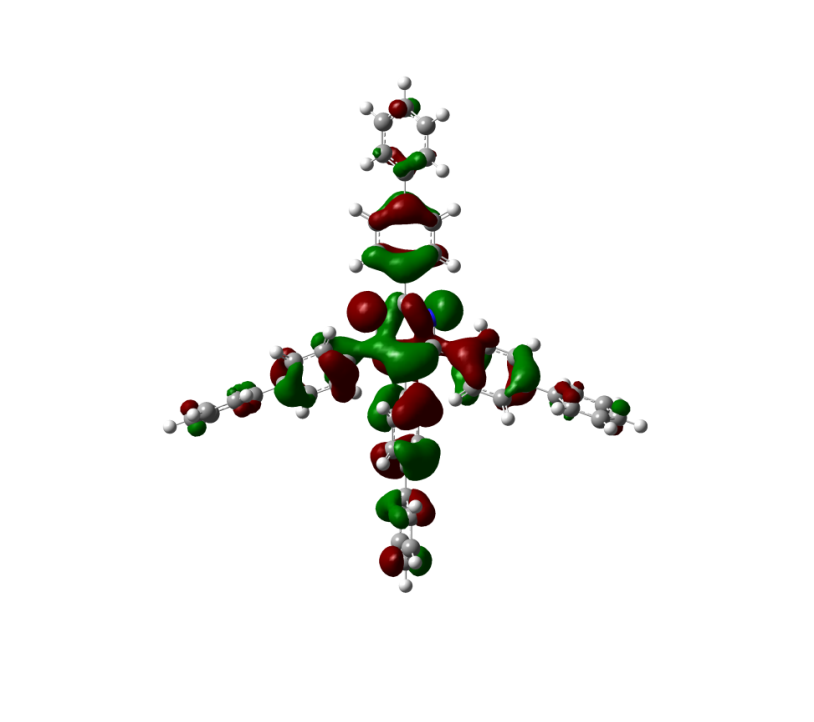 | 3.51 eV / 353 nm  *f* = 0.2791 | 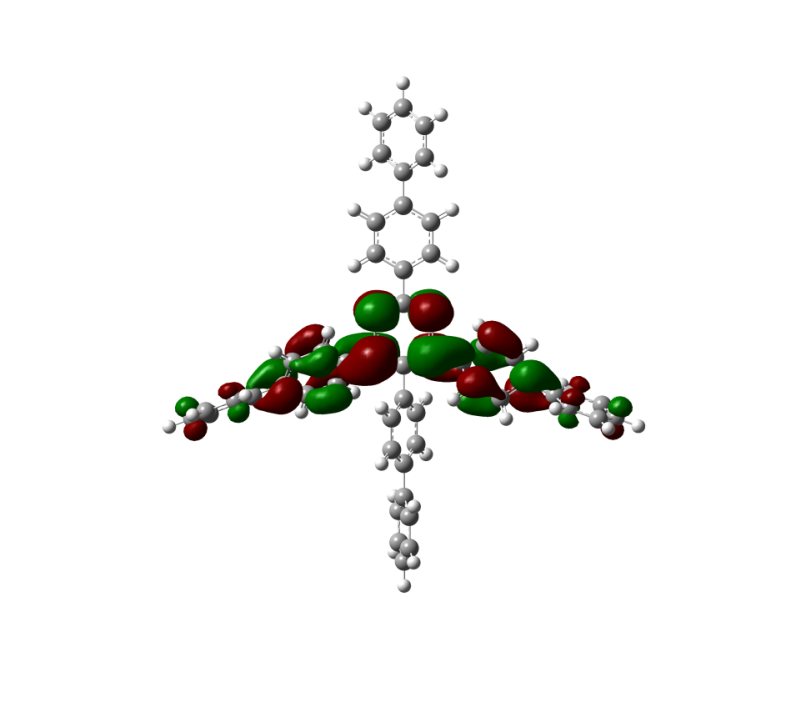 |
| S_0_–S_2_ | 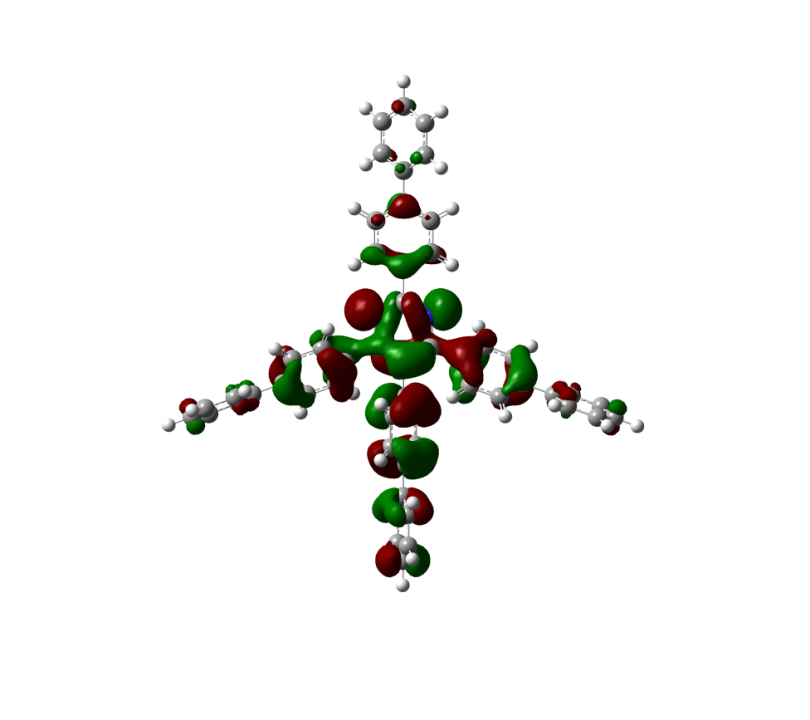 | 3.59 eV / 346 nm  *f* = 0.3643 | 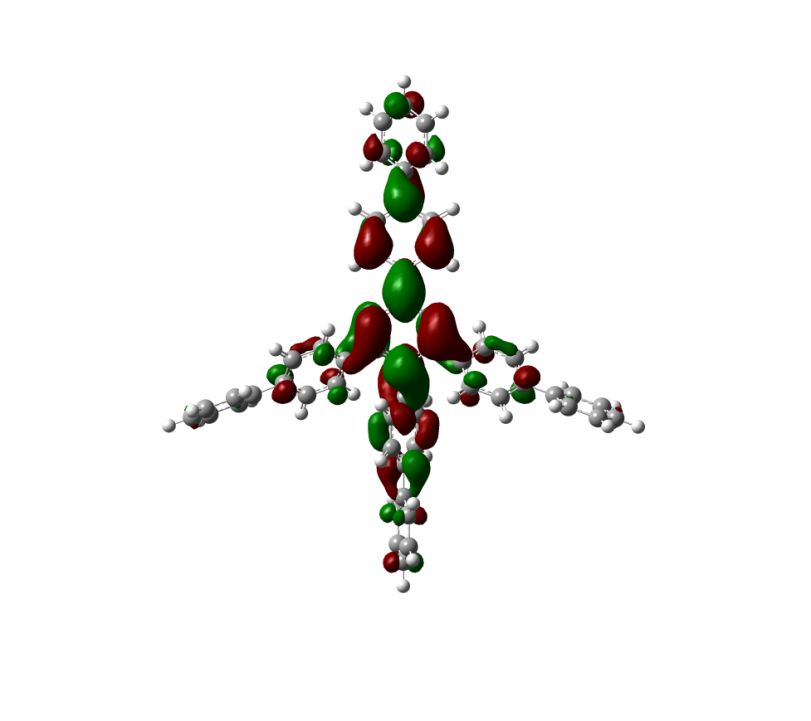 |
| S_0_–S_4_ | 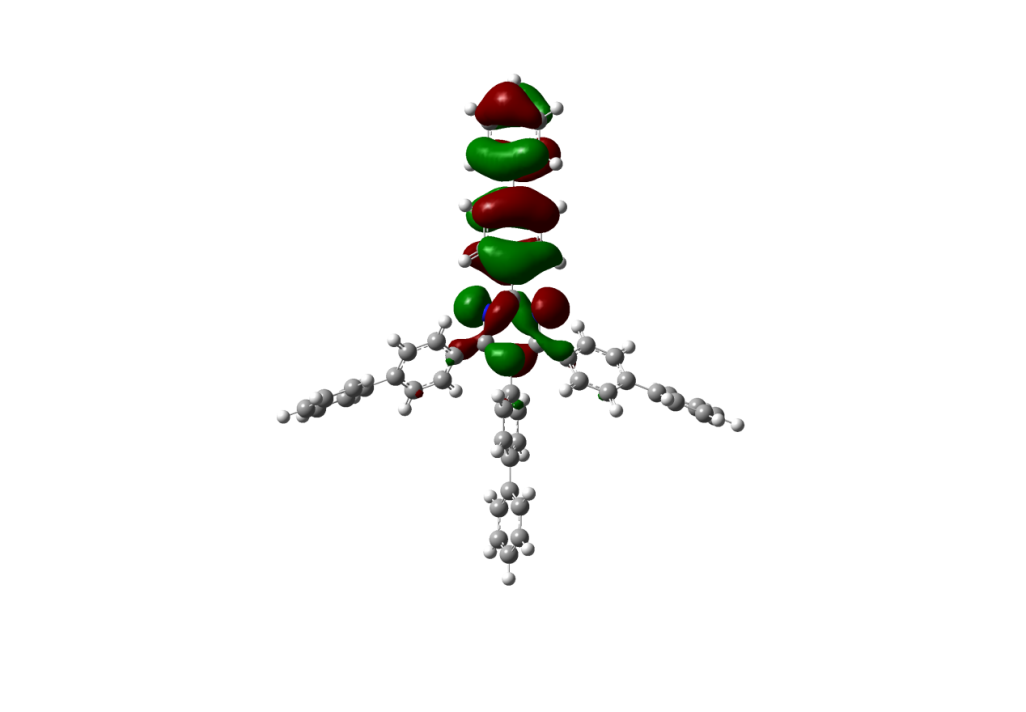 | 3.87 eV / 320 nm  *f* = 0.9193 | 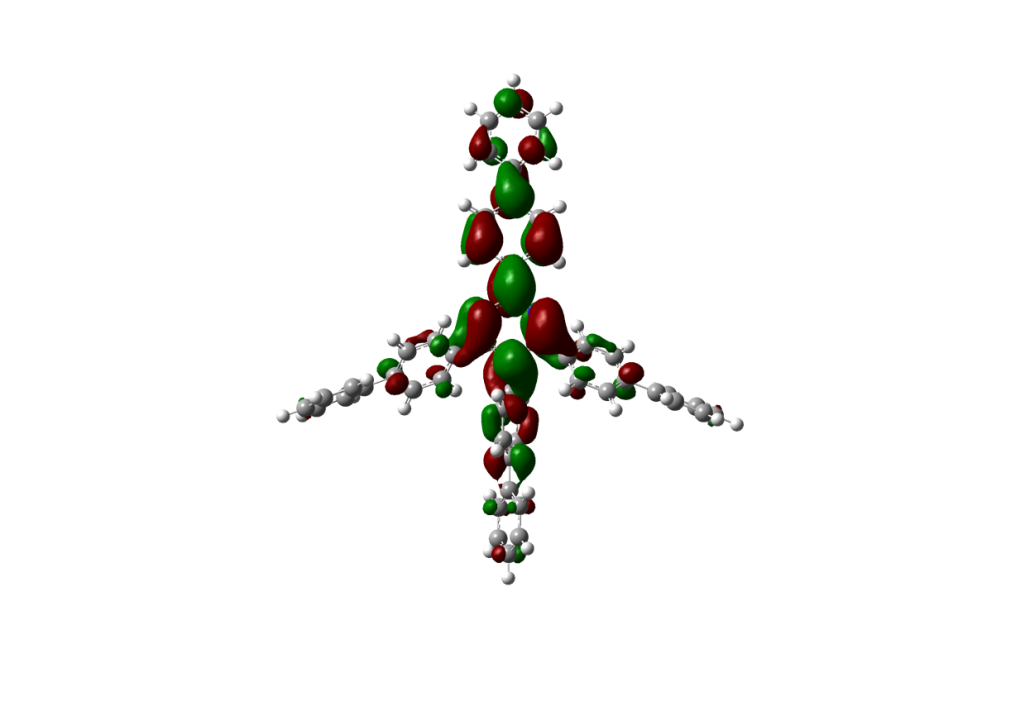 |
| S_0_–S_5_ | 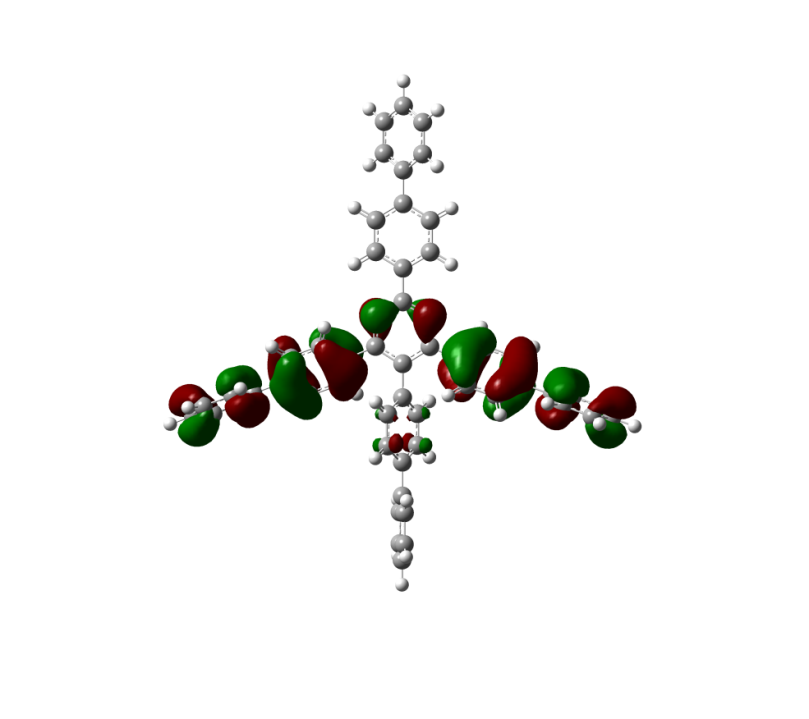 | 4.00 eV / 310 nm  *f* = 0.5485 | 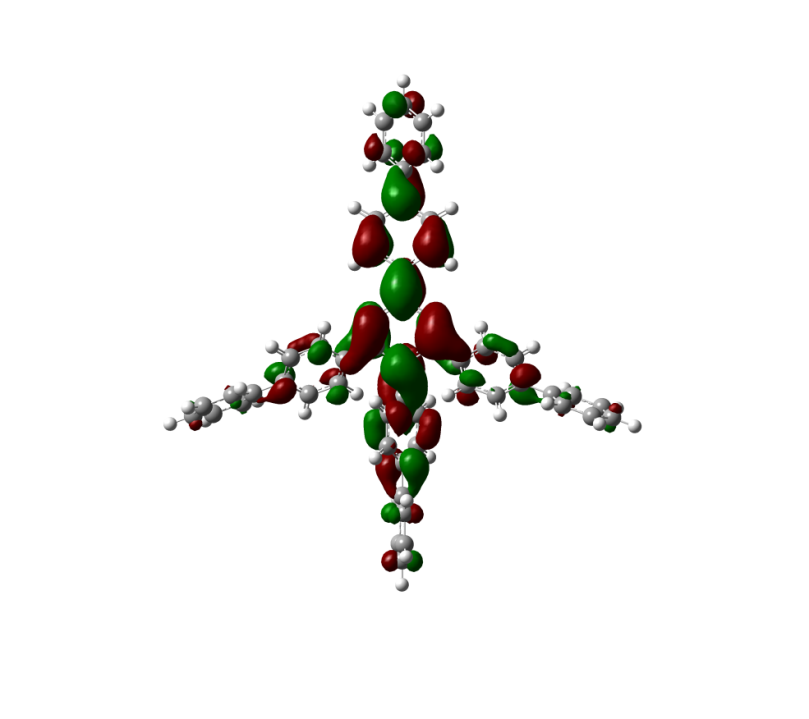 |
| S_0_–S_6_ | 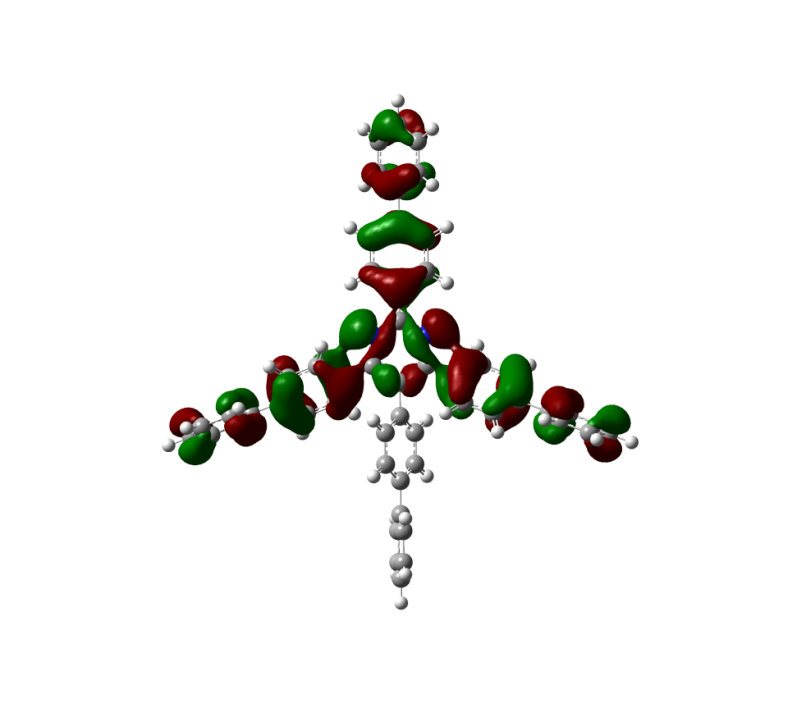 | 4.05 eV / 306 nm  *f* = 0.2448 | 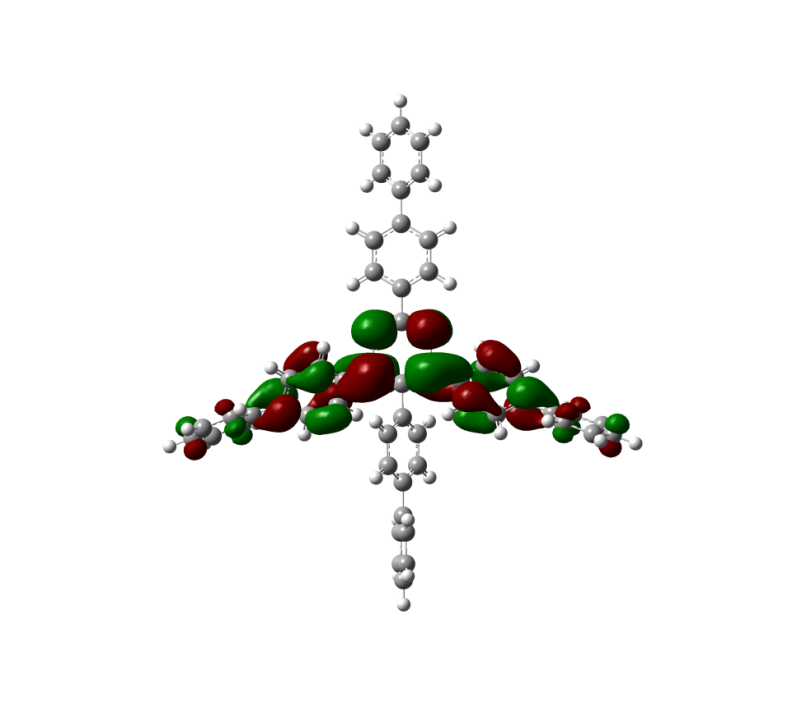 |

The absorption maximums of the three compounds mainly originate from the transition from their S_0_ states to S_4_ states. Enhanced electron push-pull effect by attaching substituents of methoxyl and phenyl groups to TPPM leads to obvious intramolecular charge transfer from the methoxyl and extra phenyl groups to the central rings in TPPM-4M and TPPM-4P after excitation, as indicated by NTO diagrams. Meanwhile, the conjugation degree is expand by the substituents, so compared to the parent one, the oscillator strengths of TPPM-4M and TPPM-4P in S_0_-S_4_ transition are increased from 0.3387 to 0.5934 and 0.9193, respectively. Furthermore, the expanded conjugation leads to more transitions with large oscillator strength between the ground state and higher excited states, thus the molar absorbance is increasingly enlarged from TPPM to TPPM-4M and TPPM-4P.

**Table S4.** Optical and thermal properties of TPPM and its derivatives.

| Compound | *λ_abs_*/nm |  | *λ_em_*/nm | |  | *Φ_F_ ^b^*/% | | |  | *T_s_ ^c^*/^o^C |
| --- | --- | --- | --- | --- | --- | --- | --- | --- | --- | --- |
|  |  |  | Soln | Solid |  | Soln | Agg*^a^* | Solid |  |  |
| TPPM | 259 |  | 390 | 398 |  | 0.4 | 1.9 | 3.7 |  | 258 |
| TPPM-4M | 290 |  | 414 | 417 |  | 2.7 | 3.9 | 8.8 |  | 345 |
| TPPM-4P | 301 |  | 411 | 414 |  | 3.6 | 5.3 | 6.2 |  | 466 |

*^a^* Aggregates are formed in THF/water mixtures with *f_w_* of 99%. *^b^ Φ_F_* is measured using Hamamatsu Quantaurus-QY C11347 spectrometer. *^c^* *T_s_* is defined as the temperature of 5% loss of weight through sublimation.

**Table S5.** Selected bond lengths (in angstrom) of TPPM and its derivatives.

|  | **TPPM** | | |  | **TPPM-4M** | | |  | **TPPM-4P** | | |
| --- | --- | --- | --- | --- | --- | --- | --- | --- | --- | --- | --- |
|  | S_0_ | S_1_ | Δ(S_1_–S_0_) |  | S_0_ | S_1_ | Δ(S_1_–S_0_) |  | S_0_ | S_1_ | Δ(S_1_–S_0_) |
| **L(N_1_**–**C_2_)** | 1.34 | 1.31 | -0.03 |  | 1.34 | 1.35 | 0.01 |  | 1.34 | 1.34 | 0 |
| **L(N_2_**–**C_4_)** | 1.34 | 1.31 | -0.03 |  | 1.34 | 1.36 | 0.02 |  | 1.34 | 1.35 | 0.01 |
| **L(N_1_**–**C_1_)** | 1.34 | 1.37 | 0.03 |  | 1.34 | 1.36 | 0.02 |  | 1.34 | 1.37 | 0.03 |
| **L(N_2_**–**C_1_)** | 1.34 | 1.37 | 0.03 |  | 1.34 | 1.32 | -0.02 |  | 1.34 | 1.32 | -0.02 |
| **L(C_2_**–**C_3_)** | 1.42 | 1.44 | 0.02 |  | 1.42 | 1.41 | -0.01 |  | 1.42 | 1.41 | -0.01 |
| **L(C_4_**–**C_3_)** | 1.42 | 1.44 | 0.02 |  | 1.42 | 1.47 | 0.05 |  | 1.42 | 1.48 | 0.06 |


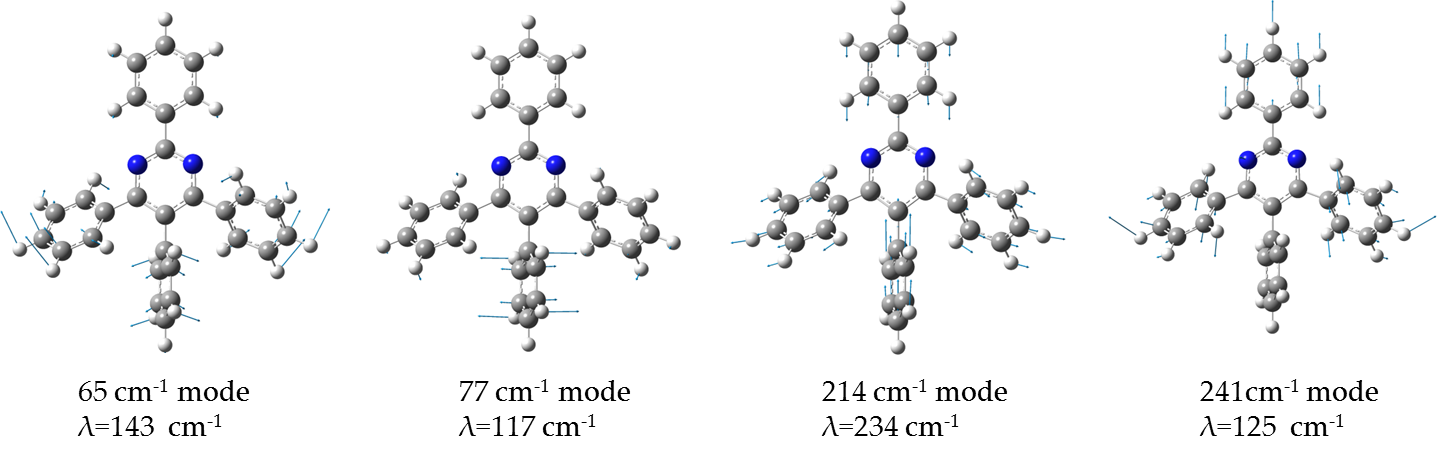


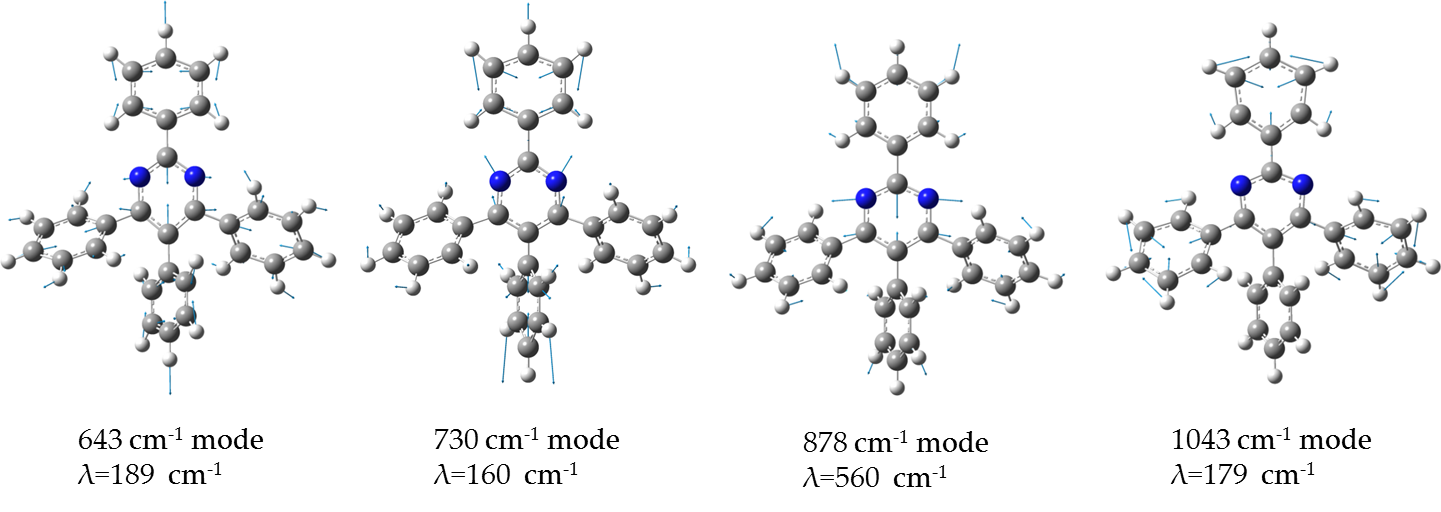


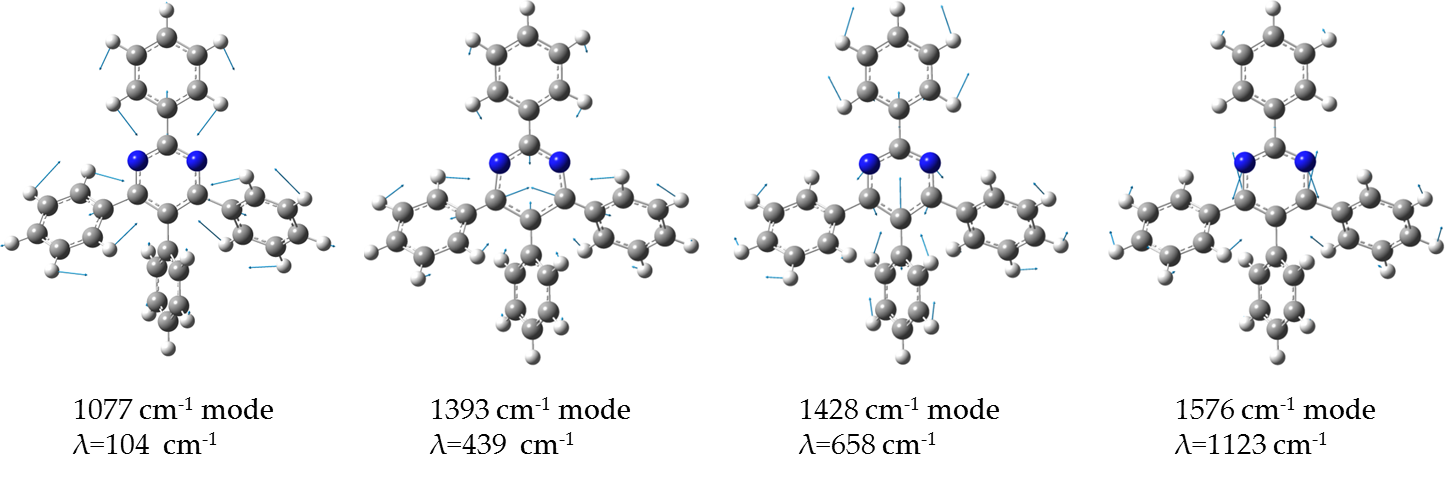


**Figure S16.** Diagrammatic illustration of selected normal modes with reorganization energy (*λ*) larger than 100 cm^-1^ of TPPM in the gas phase.

**
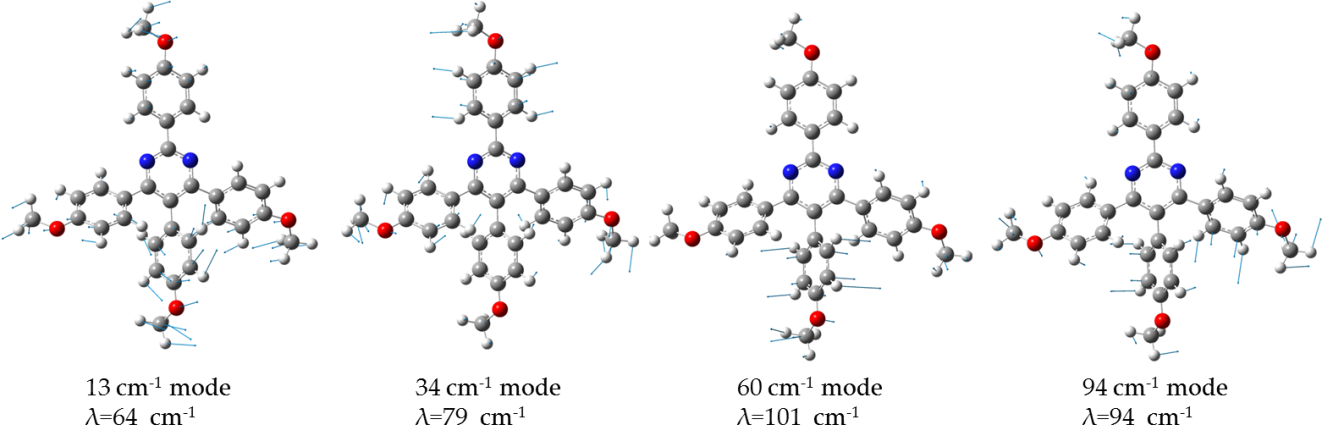
**

**
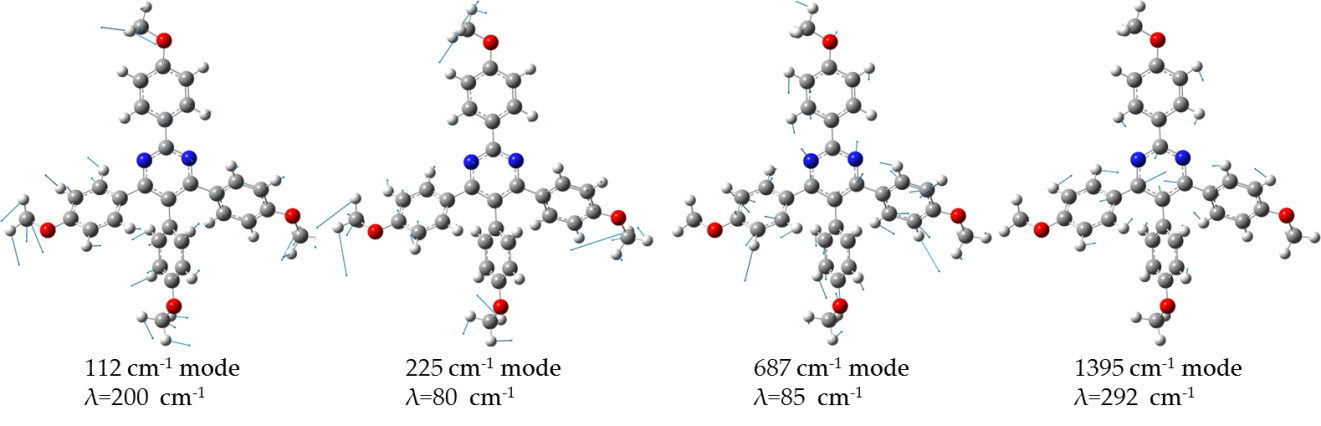
**

**
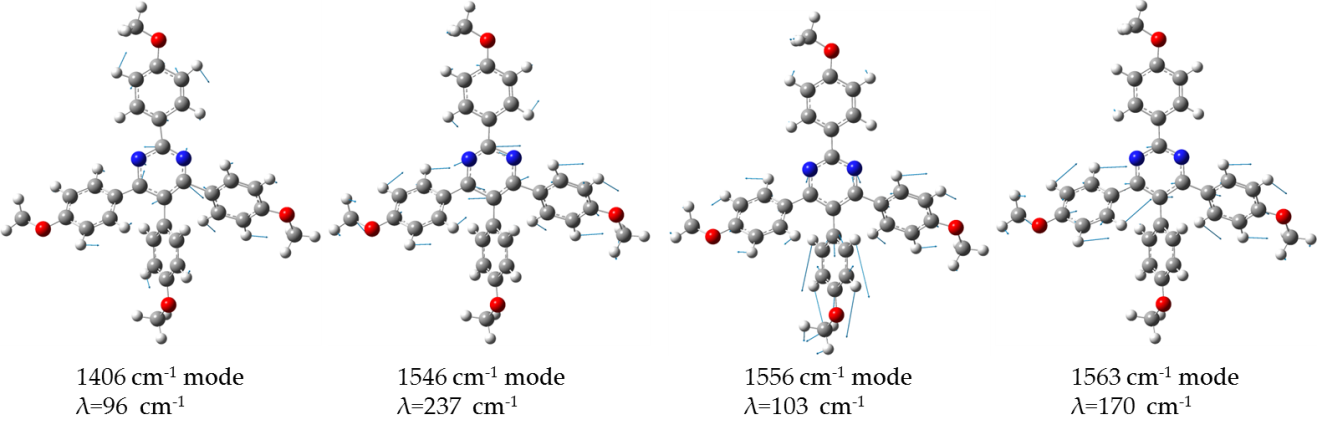
**

**Figure S17.** Diagrammatic illustration of selected normal modes with reorganization energy (*λ*) larger than 50 cm^-1^ of TPPM-4M in the gas phase.


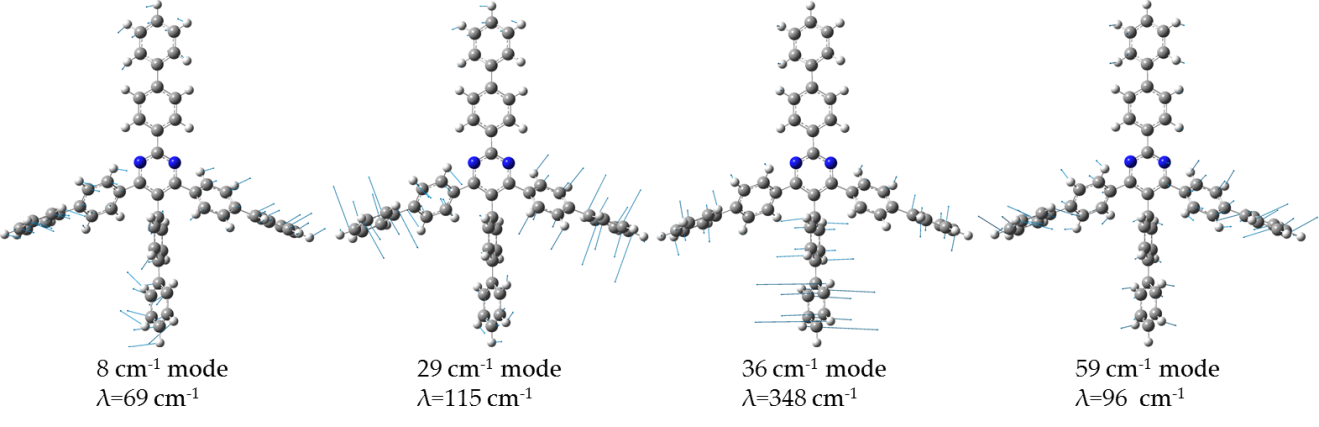


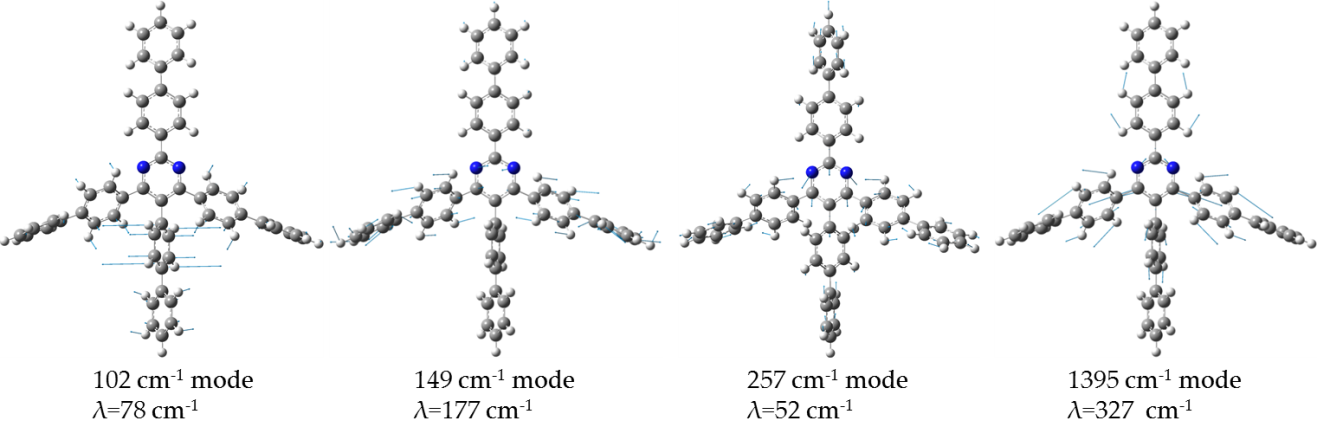


**
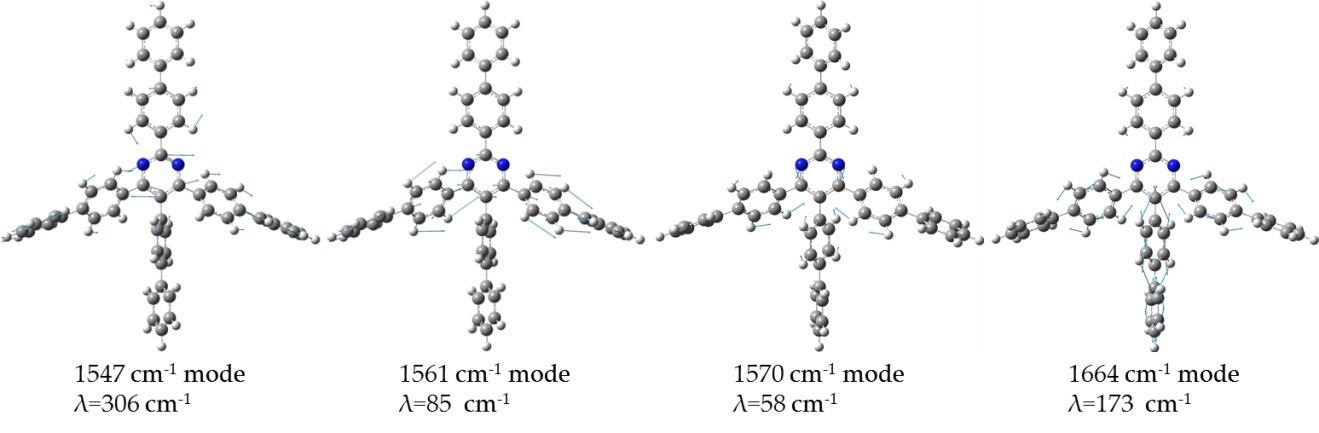
**

**Figure S18.** Diagrammatic illustration of selected normal modes with reorganization energy (*λ*) larger than 50 cm^-1^ of TPPM-4P in the gas phase.


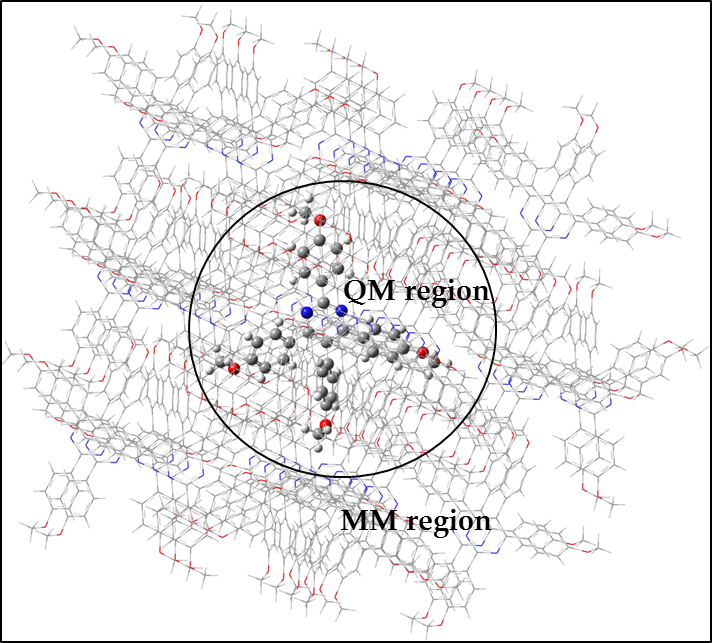


**Figure S19.** Model cluster cut from the single crystal structure for QM/MM calculation.

**Table S6.** Selected bond lengths (in angstrom) of TPPM-4M in the single crystal and corresponding parameters calculated in the gas and the crystal phase.

|  | **Single crystal** |  | **Gas phase** | | |  | **Crystal phase** | | |
| --- | --- | --- | --- | --- | --- | --- | --- | --- | --- |
|  |  |  | S_0_ | S_1_ | Δ(S_1_–S_0_) |  | S_0_ | S_1_ | Δ(S_1_–S_0_) |
| **L(N_1_**–**C_2_)** | 1.35 |  | 1.34 | 1.35 | 0.01 |  | 1.34 | 1.36 | 0.02 |
| **L(N_2_**–**C_4_)** | 1.34 |  | 1.34 | 1.36 | 0.02 |  | 1.35 | 1.37 | 0.02 |
| **L(N_1_**–**C_1_)** | 1.34 |  | 1.34 | 1.36 | 0.02 |  | 1.34 | 1.36 | 0.02 |
| **L(N_2_**–**C_1_)** | 1.34 |  | 1.34 | 1.32 | -0.02 |  | 1.34 | 1.32 | -0.02 |
| **L(C_2_**–**C_3_)** | 1.40 |  | 1.42 | 1.41 | -0.01 |  | 1.42 | 1.40 | -0.02 |
| **L(C_4_**–**C_3_)** | 1.41 |  | 1.42 | 1.47 | 0.05 |  | 1.42 | 1.47 | 0.05 |

**Table S7.** Selected bond angles (in degree) of TPPM-4M in the single crystal and corresponding parameters calculated in the gas and the crystal phase.

|  | **Single crystal** |  | **Gas phase** | | |  | **Crystal phase** | | |
| --- | --- | --- | --- | --- | --- | --- | --- | --- | --- |
|  |  |  | S_0_ | S_1_ | Δ(S_1_–S_0_) |  | S_0_ | S_1_ | Δ(S_1_–S_0_) |
| **A(C_1_**–**N_1_**–**C_2_)** | 117 |  | 118 | 117 | -1 |  | 118 | 116 | -2 |
| **A(C_1_**–**N_2_**–**C_4_)** | 118 |  | 118 | 121 | 3 |  | 118 | 122 | 4 |
| **A(N_1_**–**C_2_**–**C_3_)** | 122 |  | 122 | 123 | 1 |  | 122 | 123 | 1 |
| **A(N_2_**–**C_4_**–**C_3_)** | 121 |  | 121 | 118 | -3 |  | 121 | 116 | -5 |
| **A(N_1_**–**C_1_**–**N_2_)** | 126 |  | 125 | 125 | 0 |  | 125 | 125 | 0 |
| **A(C_2_**–**C_3_**–**C_4_)** | 116 |  | 116 | 116 | 0 |  | 116 | 117 | 1 |

**Figure S20.** PL spectrum of TPPM with addition of different amounts of PA in THF/water mixture (*f_w_* = 99%). Concentration of TPPM = 10^-5^ M. Excitation wavelength = 300 nm.

**Figure S21.** PL spectrum of TPPM-4P with addition of different amounts of PA in THF/water mixture (*f_w_* = 99%). Concentration of TPPM-4P = 10^-5^ M. Excitation wavelength = 345 nm.

**Figure S22.** PL spectrum of TPE with addition of different amounts of PA in THF/water mixture (*f_w_* = 99%). Concentration of TPE = 10^-5^ M. Excitation wavelength = 309 nm.


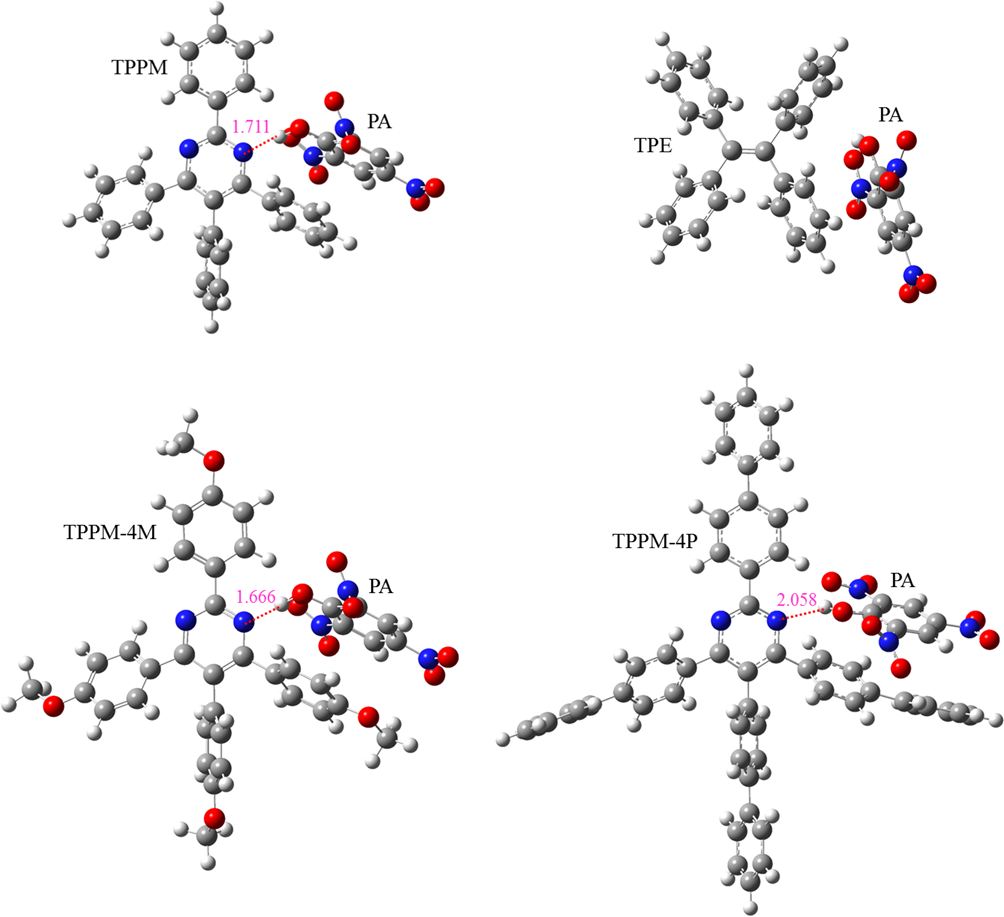


**Figure S23.** Hydrogen bonds formed among TPPM derivatives and PA molecules calculated through conformation optimization at ωb97XD/6-31G(d, p) level.

**Table S8.** Calculated lengths of hydrogen bonds and complexation energy of TPPM derivatives and PA.

| Compound | Length of H-bond (Ǻ) | Complexation energy (Kcal/mole) | Quenching Constant (M^-1^) |
| --- | --- | --- | --- |
| TPPM | 1.711 | -23.78 | 58734 |
| TPPM-4M | 1.666 | -28.43 | 41703 |
| TPPM-4P | 2.058 | -19.69 | 32269 |
| TPE | / | -11.33 | 9299 |
